# The *cisd* gene family regulates physiological germline apoptosis through *ced-13* and the canonical cell death pathway in *Caenorhabditis elegans*

**DOI:** 10.1038/s41418-018-0108-5

**Published:** 2018-04-17

**Authors:** Skylar D. King, Chipo F. Gray, Luhua Song, Rachel Nechushtai, Tina L. Gumienny, Ron Mittler, Pamela A. Padilla

**Affiliations:** 1 Department of Biological Sciences, University of North Texas, Denton, TX 76203 USA; 2 Alexander Silberman Institute of Life Sciences, Hebrew University of Jerusalem, Edmond J. Safra Campus at Givat Ram, Jerusalem, 91904 Israel; 3 Department of Biology, Texas Woman’s University, Denton, TX 76204 USA

**Keywords:** Development, Cell biology

## Abstract

Programmed cell death, which occurs through a conserved core molecular pathway, is important for fundamental developmental and homeostatic processes. The human iron–sulfur binding protein NAF-1/CISD2 binds to Bcl-2 and its disruption in cells leads to an increase in apoptosis. Other members of the CDGSH iron sulfur domain (CISD) family include mitoNEET/CISD1 and Miner2/CISD3. In humans, mutations in CISD2 result in Wolfram syndrome 2, a disease in which the patients display juvenile diabetes, neuropsychiatric disorders and defective platelet aggregation. The *C. elegans* genome contains three previously uncharacterized *cisd* genes that code for CISD-1, which has homology to mitoNEET/CISD1 and NAF-1/CISD2, and CISD-3.1 and CISD-3.2, both of which have homology to Miner2/CISD3. Disrupting the function of the *cisd* genes resulted in various germline abnormalities including distal tip cell migration defects and a significant increase in the number of cell corpses within the adult germline. This increased germ cell death is blocked by a gain-of-function mutation of the Bcl-2 homolog CED-9 and requires functional caspase CED-3 and the APAF-1 homolog CED-4. Furthermore, the increased germ cell death is facilitated by the pro-apoptotic, CED-9-binding protein CED-13, but not the related EGL-1 protein. This work is significant because it places the CISD family members as regulators of physiological germline programmed cell death acting through CED-13 and the core apoptotic machinery.

## Introduction

The core programmed cell death pathway is highly regulated to maintain normal developmental and homeostatic processes. This well-studied pathway, composed of pro-survival Bcl-2, pro-death APAF-1 and pro-death caspases, is regulated in a cell- and stimulus-dependent manner. One of the core principles regulating this pathway involves mutually exclusive interactions of Bcl-2 with the BH3 domain (Bcl-2 homology region 3) pro- or anti-apoptotic proteins that modulate Bcl-2 activity. The iron–sulfur (2Fe-2S) protein NAF-1/CDGSH iron sulfur domain 2 (CISD2; nutrient autophagy factor) was identified as a protein that binds to Bcl-2 at the ER [1]. Additionally, the displacement of NAF-1 from Bcl-2 binding occurs via the Endoplasmic Reticulum (ER) localized BH3-only protein Bik [1]. These results led to the hypothesis that the Bcl-2:NAF-1 complex at the ER plays a key role in regulating apoptosis and autophagy in mammalian cells [2–4]. Mapping of the binding interface between Bcl-2 and NAF-1 subsequently revealed that NAF-1 binds to Bcl-2 at the same site that a BH3-only protein would [5]. Further support for the involvement of NAF-1 in autophagy/apoptosis regulation comes from studies in cancer cell lines, xenograft tumors and the null NAF-1 mouse model, in which NAF-1 dysfunction led to the activation of apoptosis [5]. However, the role NAF-1 has in programmed cell death and whether this protein regulates apoptosis via binding to Bcl-2 is not understood [1, 3, 4].

The CISD protein family includes NAF-1/CISD2, the mitochondrial outer membrane protein mitoNEET/CISD1 and the mitochondrial protein CISD3/Miner2 [6–9]. Phenotype analyses indicate that the NAF-1/CISD2 and mitoNEET/CISD1 proteins are multifunctional and have various cellular roles including those involved in mitochondria function and cell proliferation [6, 10–12]. In humans, a mutation in CISD2 results in Wolfram syndrome 2 disease, an autosomal recessive disorder characterized by early onset of juvenile diabetes mellitus, optic atrophy, deafness, renal abnormalities and neuropsychiatric disorders [13–19]. Relative to mitoNEET and NAF-1, much less is understood about the function of Miner2/CISD3 because in-depth phenotype analysis associated with CISD3 gene dysfunction has not been conducted.

Many biological processes, including apoptosis, have been elucidated from the genetic and cell biological analyses in the *Caenorhabditis elegans* (*C. elegans*) model system [20, 21]. Here we use the *C. elegans* genetic system to study the function of the *cisd* gene family. The *C. elegans* genome contains three previously uncharacterized *cisd* genes (*cisd-1*, *cisd-3.1* and *cisd-3.2*). The *cisd-1* gene codes for a protein that shows homology to the vertebrate mitoNEET/CISD1 and NAF-1/CISD2, and the *cisd-3.1* and *cisd-3.2* genes code for proteins that show homology to vertebrate CISD3 [9]. Using CRISPR technology, we developed a *cisd-1p::GFP* reporter and determined that the *cisd-1* promoter drives expression in the hermaphrodite germline. The *C. elegans* hermaphrodite gonad is a U-shaped structure that consists of somatic cells and the germline, which produces sperm and oocytes [22]. During the process of oogenesis, some cells will naturally undergo apoptosis; this process is referred to as germline physiological apoptosis [20]. The analysis of germline physiological apoptosis within *C. elegans* provides a model for cell death mechanisms needed to maintain the structure and function of an organ. In addition to physiological cell death, apoptosis within the germline will increase in response to various stresses (e.g., DNA damage) or defects in synapsis during meiosis [20, 23]. The mechanisms involved in physiological cell death and environmentally induced cell death within the *C. elegans* germline involve the core apoptotic machinery (CED-9, CED-4 and CED-3) [24]. Although many pioneering studies have been done using *C. elegans* to reveal the genetic mechanisms regulating apoptosis, questions still remain regarding the regulation of physiological germline apoptosis [20, 25, 26].

Here, we determined that disruption of *cisd-1* gene function, using RNA interference (RNAi), or mutant alleles [*cisd-1(tm4993)* deletion mutant*, cisd-1(pnIs27)* insertion mutant] led to germline abnormalities including an increase in the number of cell corpses and distal tip cell migration defects. RNAi knock-down of *cisd-3.1* or *cisd-3.2* resulted in similar phenotypes indicating functional overlap within the *cisd* gene family. Disruption of *ced-3*/caspase or *ced-4*/APAF-1 function, or the gain-of-function *ced-9(n1950)* mutation reduced the number of cell corpses in the germline of the *cisd-1(tm4993)* animal. Additionally, disruption of *ced-13*, which codes for a pro-apoptotic, BH3 (Bcl-2 homology region 3) protein that physically interacts with the anti-apoptotic CED-9/Bcl-2 protein, significantly reduced the number of germ cell corpses observed in the *cisd-1(tm4993)*, *cisd-3.1(RNAi*) and *cisd-3.2(RNAi)* animals. These results show that the *C. elegans cisd* genes function in the process of apoptosis and place the CISD protein family as regulators of physiological germline programmed cell death with CED-13.

## Results

### Disruption of the *cisd* genes impact germline structure and germ cell survival

**Fig. 1:**
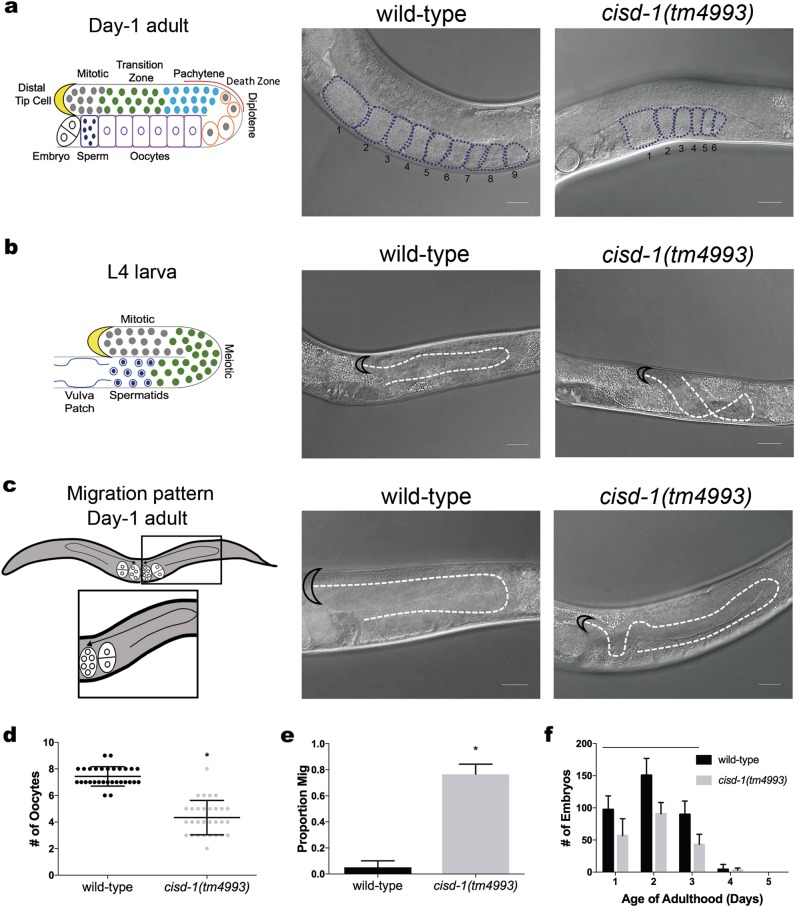
The *cisd-1(tm4993*) animals displays germline abnormalities. **a** Left: schematic diagram of the *C. elegans* adult hermaphrodite gonad; shown is one of two gonads to note the meiotic regions, cell death zone, and location of the distal tip cell (DTC) [20, 61]. Right: representative images of the gonad arm for the N2 wild-type or the *cisd-1(tm4993*) 1-day old adult hermaphrodite. The oocytes are outlined and the number of oocytes present in the gonad are represented. **b** Left: Schematic diagram of the *C. elegans* L4 larvae gonad; shown is one of two gonads to note the gonad structure and location of the DTC. Right: representative images of the gonad arm for the N2 wild-type or the *cisd-1(tm4993*) L4 larvae hermaphrodite. The *cisd-1(tm4993*) animal displays a DTC migration (Mig) defect. The dashed line indicates the distal tip cell migration pattern and black half-moon outlines the DTC. **c** Left: schematic diagram of a 1-day old adult hermaphrodite showing the migration pattern of the DTC (black arrows). Right: representative images of the gonad arm for the N2 wild-type or the *cisd-1(tm4993*) 1-day old adult hermaphrodite. The *cisd-1(tm4993*) animal displays a DTC migration (Mig) defect. The dashed line indicates distal tip cell migration pattern and black half-moon outlines the DTC. For **a**, **b** and **c**, the scale bar = 20 µm. **d** There is a significant decrease in the number of maturing oocytes in the gonad of *cisd-1(tm4993)* one-day old adult hermaphrodite relative to the N2 wild-type (* indicates *P* < 0.005, two-tailed unpaired *t*-test). Three independent experiments for a total of 30 animals were analyzed. **e** There is a significant increase in the proportion of animals that displayed a Mig phenotype in the *cisd-1(tm4993)* 1-day old adult hermaphrodite gonad relative to N2 wild-type (* indicates *P* < 0.0005, two-tailed unpaired *t-*test). **f** The average number of progeny produced in the 1- to 5-day old *cisd-1(tm4993)* adult relative to the N2 wild-type of the same developmental stage. There is a significant decrease in the number of progeny produced by the 1- to 3-day old *cisd-1(tm4993)* adult relative to N2 wild-type adults (bar indicates *P* < 0.05, two-way ANOVA, Sidak’s multiple comparisons test). For **d**, **e** and **f**, error bar equals standard deviation.

The *C. elegans* genome contains three previously uncharacterized *cisd* genes (*cisd-1*, *cisd-3.1* and *cisd-3.2*) that code for the CISD proteins (Figure S1a). The *cisd-1* gene codes for two isoforms (Figure S1b) that show homology to the vertebrate mitoNEET/CISD1 and NAF-1/CISD2 (Figure S1c), and the *cisd-3.1* and *cisd-3.2* genes code for proteins that show homology to vertebrate CISD3 (Figure S1d) [9]. All three of the CISD proteins contain putative 2Fe-2S domain(s) with their signature CDGSH motif (Figure S1a). To study the *cisd-1* gene function in the context of a whole organism, we used the *C. elegans* genetic mutant *cisd-1(tm4993)*. The *cisd-1(tm4993)* mutant contains a 316 basepair (bp) deletion that removes a significant portion of the coding region of both *cisd-1a* and *cisd-1b*. The *cisd-1* mRNA is not detected, by quantitative reverse transcriptase-PCR (qRT-PCR), in the *cisd-1(tm4993)* animal indicating that the *tm4993* allele is a null mutation (Figure S2a). The *cisd-1(tm4993)* animals displayed various germline defects (Fig. 1). First, the number of differentiated oocytes is reduced in the *cisd-1(tm4993)* mutant relative to N2 wild-type animals (Figs. 1a, d). Additionally, the majority of *cisd-1(tm4993)* animals display a germline distal tip cell migration defect (referred to as a Mig phenotype) [27]. The Mig phenotype is observable in L4 larvae (Fig. 1b) and young 1-day old adults (Figs. 1c, e). The reduced number of oocytes and increased Mig phenotype was also observed within the germline of *cisd-1(RNAi)* animals (Figure S3a, b). Note that RNAi effectively reduced *cisd-1* gene function as determined by qRT-PCR (Figure S2b). The germline defects associated with the *cisd-1(tm4993)* allele do not lead to complete sterility but there is a reduction in the number of eggs laid relative to N2 control (Fig. 1f). The total number of offspring for the *cisd-1(tm4993)* hermaphrodite relative to the N2 control is significantly lower (*cisd-1(tm4993)* = 193.8 ± 17.7, N2 = 341.8 ± 15.8, *P* < 0.001, two-tailed unpaired *t*-test). Combined, these data support the idea that *cisd-1* function is important for normal germline function.

**Fig. 2:**
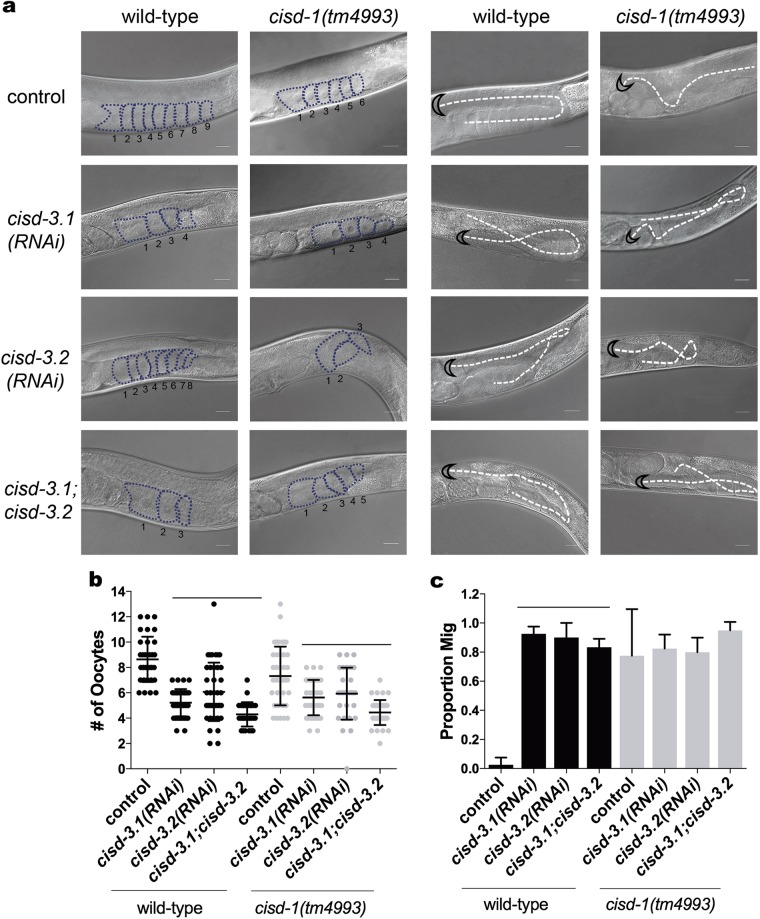
Disruption of the *cisd*-3.1 and *cisd-3.2* gene functions lead to germline defects. **a** Representative images of one gonad arm in *cisd-3.1(RNAi)*, *cisd-3.2(RNAi)*, or *cisd-3.1(RNAi)*; *cisd-3.2(RNAi)* in N2 wild-type or *cisd-1(tm4993)* 1-day old adult hermaphrodites. The RNAi control is when the respective genotype is fed HT115 bacteria with empty vector. The knock-down of the *cisd-3.1* and/or *cisd-3.2* gene function results in germline defects. Defects include abnormal oocyte number (left panel) and DTC migration (Mig) defects (right panel). The oocytes are outlined and the number of oocytes present in the gonad are represented. The dashed line indicates the DTC migration pattern and the black half-moon outlines the DTC. Scale bar = 20 μm. **b** There is a significant decrease in the number of maturing oocytes in the gonad arm of *cisd-3.1(RNAi)*, *cisd-3.2(RNAi)* and *cisd-3.1(RNAi); cisd-3.2(RNAi)* animal relative to the respective control animal (N2 wild-type, black symbol or *cisd-1(tm4993)*, gray symbol on scatter plot), (one-way ANOVA, Dunnett multiple comparison test, bar indicates *P* ≤ 0.05). **c** There is a significant increase in DTC migration defects in the *cisd-3.1(RNAi), cisd-3.2(RNAi)*, and *cisd-3.1(RNAi); cisd-3.2(RNAi)* animal relative to N2 wild-type (one-way ANOVA, Dunnett multiple comparison test, bar indicates *P* < 0.05). For **b** and **c**, at least 30 animals from three independent experiments were examined; error bar equals standard deviation.

**Fig. 3:**
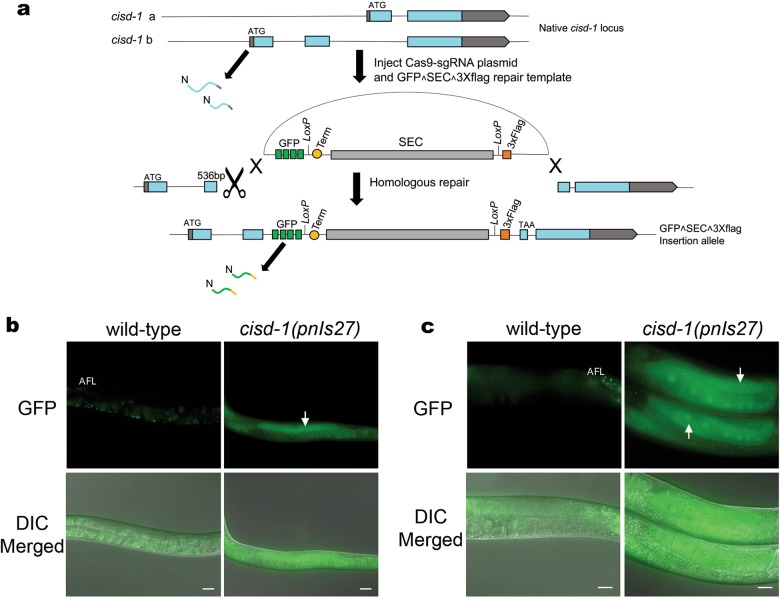
The *cisd-1* gene is expressed in the germline. **a** Illustration of the *cisd-1* locus predicted to produce two isoforms from the *cisd-1a* and *cisd-1b* transcripts. CRISPR editing was used to produce a GFP transcriptional reporter strain (PM152) that disrupts the *cisd-1* gene. The GFP^SEC^3xFlag sequence was inserted into the second exon of *cisd-1b* at 536 bp from the start site. **b** Representative images of L4 *cisd-1*(*pnls27*) animals and **c** 1-day old adult *cisd-1*(*pnls27*) animals exhibiting GFP expression in the gonad arm. The N2 wild-type animal is shown to communicate the typical autofluorescence (AFL) observed within the intestine of *C. elegans*. White arrows indicate transcriptional GFP expression in the germline of *cisd-1(pnls27)* animals. For **b** and **c**, the top panel shows fluorescent images and the bottom panel shows a fluorescent image merged with the DIC image; scale bar = 20 µm. Images are representative animals from 30 animals examined.

Relative to mitoNEET and NAF-1 in mammalian systems, much less is understood about the function of Miner2/CISD3. Here, we used RNAi to knock-down *cisd-3.1* and/or *cisd-3.2* gene function, in both the N2 wild-type and *cisd-1(tm4993)* animals to determine if such impacts the germline structure and if the phenotype is more severe when more than one *cisd* gene is dysfunctional. Knock-down of the *cisd-3.1* and/or *cisd-3.2* gene function reduced the number of differentiated oocytes within the germline of 1-day old adults, relative to N2 wild-type control animals (Figs. 2a (left panel), b). The combination of *cisd-3.1* and/or *cisd-3.2* knock-down with the *cisd-1(tm4993)* mutation further reduced the number of mature oocytes relative to the *cisd-1(tm4993)* single mutant (Fig. 2b, gray dot plot). The disruption of *cisd-3.1* or *cisd-3.2* gene function also resulted in a Mig phenotype (Figs. 2a (right panel), c). Relative to the *cisd-1(tm4993)*, the combination of *cisd-3.1* and/or *cisd-3.2* knock-down with the *cisd-1(tm4993)* mutation did not further increase the number of animals displaying the Mig phenotype relative to the *cisd-1(tm4993)* (Fig. 3c, gray bars); this is likely due to the fact that the penetrance of the Mig phenotype is already high when a *cisd* gene is disrupted. These results indicate that the *cisd* gene family has a role in germline structure and function and the *cisd* genes have overlapping functions. Note that RNAi effectively reduced *cisd-3.1* and *cisd-3.2* gene function as determined by qRT-PCR (Figure S2b).

Our results indicate that *cisd* dysfunction leads to germline defects. Thus, we wanted to examine if *cisd-1* is expressed within the germline. Transgenic genes are known to be silenced within the germline [28]. However, recently developed CRISPR methods have been shown to be effective in producing GFP transcriptional reporters for genes known to be expressed within the germline [29]. We used this approach to produce the *cisd-1(pnls27* [GFP^SEC^3xFlag::*cisd-1*]) transcriptional reporter strain (PM154) (Fig. 3a). This methodological approach also produces an insertion mutant, which can be used to verify phenotypes associated with *cisd-1* dysfunction (Fig. 3a) [29]. The *cisd-1* mRNA, as detected by qRT-PCR analysis, is significantly reduced in the *cisd-1(pnIs27)* mutant suggesting this allele is a null mutation (Figure S2a). The *cisd-1(pnIs27)* animals showed GFP expression in the germline of L4 (Fig. 3b) and young adults (Fig. 3c). The GFP expression was also observed in embryonic blastomeres, and the muscle and intestinal cells within larvae and young adults (data not shown). Similar to the *cisd-1(tm4993)* mutant, the *cisd-1(pnIs27)* animals had a reduced number of mature oocytes (Figure S3c) and a reduction in progeny relative to control N2 wild-type animals (Figure S3d). These data provide further support that CISD-1 functions within the *C. elegans* germline.

### Reduction of *cisd* gene family function increases cell corpses in the germline

**Fig. 4:**
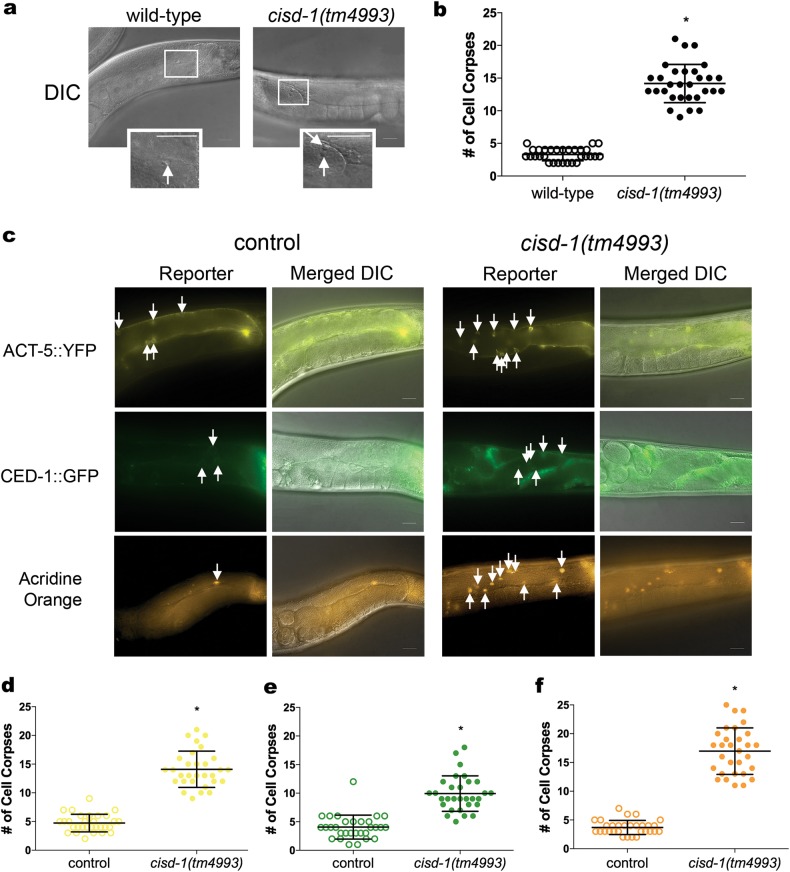
The *cisd-1(tm4993)* animals exhibits an increase in germline cell corpses. **a** Representative DIC images of one gonad arm with cells undergoing apoptosis in the *cisd-1(tm4993)* and N2 wild-type animal. The boxed region shows an enlarged area containing the cell corpse(s); the white arrow points to an apoptotic cell appearing as a “button”. Scale bar = 20 µm. **b** The *cisd-1(tm4993)* animal has a significantly higher number of apoptotic cells relative to the N2 wild-type as determined by analysis of the gonad using DIC microscopy. **c** Representative fluorescent microscopy images of the germline for control or *cisd-1(tm4993)* animals. Reporters were used to mark specific aspects of programmed cell death (ACT-5::YFP, CED-1::GFP, or acridine orange). Shown are the fluorescent reporters individually or merged with the DIC image to better visualize the gonad anatomy. White arrows mark either germ cell corpses with surrounding actin bundles in the early stages of apoptosis (ACT-5::YFP), surrounding cells that have initiated engulfment (CED-1::GFP), or cells positive for cell surface acridine orange staining. Scale bar = 20 µm. **d-f** Relative to control populations, the *cisd-1(tm4993)* animals have a significantly higher number of germline apoptotic cells marked by **d** ACT-5::YFP, **e** CED-1::GFP positive cells, or **f** acridine orange. For **b**, **d**, **e** and **f**, the number of apoptotic corpses within the gonad was quantified in animals from three independent experiments for a total of at least 30 animals (*indicates *P* < 0.0001, two-tailed unpaired parametric *t*-test, or Mann–Whitney two-tailed nonparametric test). The error bar indicates standard deviation.

Previously we showed that breast cancer cell lines with suppressed NAF-1/CISD2 expression (grown in culture or xenograft tumors) display features of activated apoptosis [30]. Furthermore, through the use of synthetic peptides the NAF-1/CISD2 protein was shown to interact with Bcl-2 [5]. However, it is unclear what role NAF-1/CISD2 has in the apoptotic pathway. Given that the knock-down of *cisd* function in *C. elegans* causes germline defects, including the Mig phenotype, which is associated with mutations in genes that have a role in cell corpse engulfment following germline apoptosis (e.g., *ced-5*, *ced-10*) [31–33], we asked if the *cisd* genes function during the physiological germline programmed cell death process in *C. elegans*. Using differential interference contrast (DIC) microscopy to directly visualize cell corpses, we determined that the *cisd-1(tm4993)* (Figs. 4a, b) and *cisd-1(pnls27)* (Figure S3e) animals have an increased number of cell corpses in the germline relative to control wild-type animals. To confirm and further examine this finding, we used established programmed cell death reporters that mark distinct time points in the process of programmed cell death [34]. The ACT-5::YFP marker is expressed in somatic sheath cells and marks pre-disc corpses; this is used as a reporter for the early stages of apoptosis [35]. The CED-1::GFP marker is expressed in cells that have initiated the process of engulfment [36]. The vital dye acridine orange stains internalized apoptotic cells [24, 37]. Using these three different approaches, we confirmed that the *cisd-1(tm4993)* animal has a significantly higher number of cell corpses in the germline relative to respective controls (Figs. 4c–f). Animals defective in cell corpse engulfment (e.g., *dyn-1(ky51)*, *ced-1(e1735)*, and *vps-34(RNAi)*) have gonads that accumulate cell death corpses when visualized by DIC microscopy but are defective for acridine orange staining indicating that these genes have a functional role in cell corpse processing [20, 37]. Within the gonad of the *cisd-1(tm4993)* animal, the cell corpses, as visualized by DIC microscopy, were positive for acridine orange suggesting that CISD-1 is not involved with cell corpse processing (Figs. 4c, f). To confirm that the *cisd-1* mutant does not display cell corpse processing defects, we assayed for the persistence of cell corpses in newly hatched *cisd-1(tm4993)* L1 larvae and embryos at the bean or comma stage of development, relative to N2 wild-type and the *ced-1(e1735)* positive control animal. The N2 wild-type and *cisd-1(tm4993)* L1 larvae did not contain cell corpses that persisted into the L1 larvae stage (30 animals assayed), whereas the positive control *ced-1(e1735)* animal did show the persistent cell corpse phenotype. Additionally, there was not a significant difference between the number of cell corpses observed within the N2 wild-type and *cisd-1(tm4993)* comma-staged embryo (12.1 ± 1.3, 12.1 ± 1.0, *P* > 0.05); the positive control *ced-1(e1735)* embryo did have a significantly higher number of cell corpses as expected (20.0 ± 2.4, *P* < 0.001). Together, these data indicate that the *cisd-1(tm4993)* mutant has a defect in the regulation of physiological apoptosis and that this defect is not associated with a defect in the cell corpse engulfment process.

**Fig. 5:**
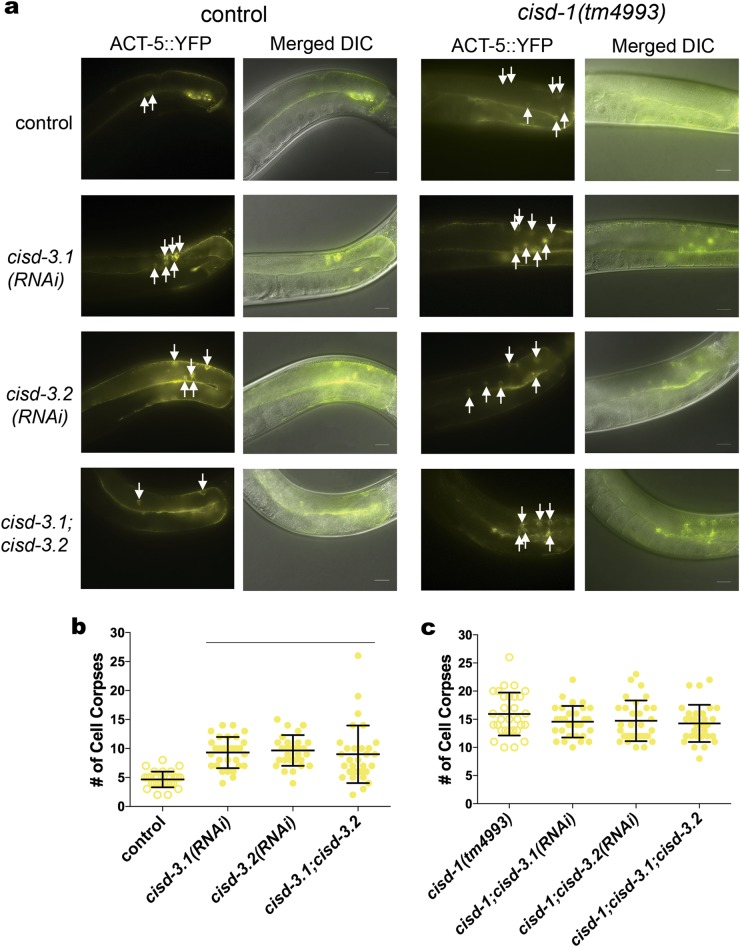
The *cisd-3.1(RNAi)* and *cisd-3.2(RNAi)* animals exhibits an increase in germ cell corpses. **a** Representative images of the gonad arm of *cisd-3.1(RNAi)*, *cisd-3.2(RNAi)* or *cisd-3.1(RNAi)*; *cisd-3.2(RNAi)* in the background of control animal (left panel) or the *cisd-1(tm4993)* animal (right panel). The ACT-5::YFP fluorescent reporter is shown individually or merged with the DIC image. White arrows point to apoptotic cells within the gonad. Scale bar = 20 µm. **b** The *cisd-3.1(RNAi)*, *cisd-3.2(RNAi)* and *cisd-3.1(RNAi)*; *cisd-3.2(RNAi)* animals have a significantly higher number of cell death corpses relative to the control animal (bar indicates *P* < 0.001, Kruskal–Wallis test, Dunn’s multiple comparisons test). **c** The *cisd-3.1(RNAi); cisd-1(tm4993)*, *cisd-3.2(RNAi); cisd-1(tm4993)* or *cisd-3.1(RNAi)*; *cisd-3.2(RNAi); cisd-1(tm4993)* animals do not have a significantly higher number of cell death corpses relative to *cisd-1(tm4993)* animals (one-way ANOVA, Dunnett multiple comparison test). For **b** and **c**, the number of apoptotic corpses within the gonad were quantified in animals from three independent experiments for a total of at least thirty animals. Error bar represents standard deviation.

We used the ACT-5::YFP and CED-1::GFP reporters to examine if knock-down of *cisd-3.1* and/or *cisd-3.2* function impacts physiological programmed cell death within the germline. The *cisd-3.1(RNAi), cisd-3.2(RNAi)*, and *cisd-3.1(RNAi); cisd-3.2(RNAi)* animals had an increased number of cell corpses within the germline relative to control (Figs. 5a (left panel), b; Figure S4a left panel, b). The combination of *cisd-3.1* and/or *cisd-3.2* knock-down with the *cisd-1(tm4993)* mutation did not further increase in the number of cell corpses relative to *cisd-1(tm4993)* (Figs. 5a (right panel), c; Figure S4a right panel, c). Our data support the idea that the *cisd* gene family functions in the process of physiological germline apoptosis.

### The increased germline cell death in the *cisd-1* mutant is mediated through the core apoptotic machinery and depends on CED-13 function

**Fig. 6:**
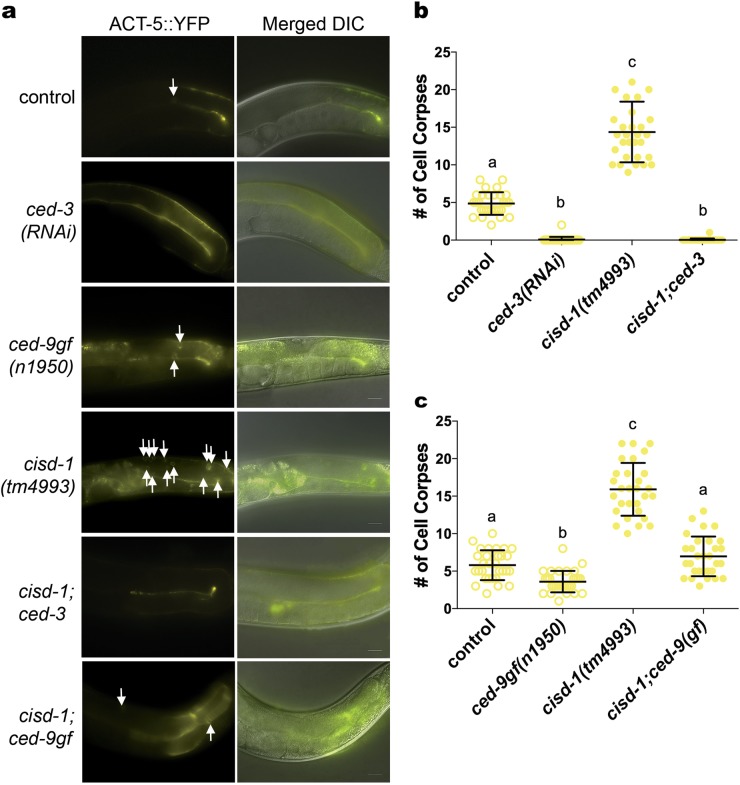
The number of gonad cell corpses is reduced in the *cisd-1(tm4993)* animals with disrupted apoptotic core machinery processes. The number of apoptotic germ cells within the gonad was visualized and quantified, using the ACT-5::YFP reporter strain, for the respective animals: control, *ced-3(RNAi)*, *ced-9(n1950gf), cisd-1(tm4993)*, *cisd-1(tm4993); ced-3(RNAi)* and *cisd-1(tm4993); ced-9(n1950gf))*. **a** Representative images of the gonad arm in respective animals. The ACT-5::YFP reporter is shown individually and merged with the DIC image. White arrows point to the apoptotic cells within the gonad. Scale bar = 20 µm. **b** The knock-down of *ced-3* using RNAi reduced the number of cell corpses in the gonad of *cisd-1(tm4993)* animals. Relative to control animals, the number of cell death corpses within the gonad was significantly reduced in the *ced-3(RNAi)* animals, indicating effective RNAi of *ced-3*. **c** The *ced-9(n1950gf)* allele reduced the number of cell corpses in the gonad of *cisd-1(tm4993)* animals. **b, c** Identical letters indicate groups with no significant differences; different letters indicate *P* < 0.05 (Kruskal–Wallis, Dunn’s multiple comparison test). The number of apoptotic corpses within the gonad was quantified in animals from three independent experiments for a total of at least 30 animals. Error bar represents standard deviation.

The core apoptotic machinery in *C. elegans* is coded by the *ced-3*, *ced-4* and *ced-9* genes that are required for the regulation of programmed cell death during embryogenesis and within the germline [20, 26]. The *ced-3* gene encodes a caspase, the *ced-4* gene is orthologous to the APAF-1 gene encoding the apoptotic protease-activating factor 1 (Apaf-1) and the *ced-9* gene is orthologous to the BCL-2 gene encoding the apoptotic regulator (Bcl-2). The CED-9 protein functions to prevent cells from dying by binding and regulating CED-4; this will ultimately impact the ability for CED-3 to cleave cellular proteins. We examined if the increased germline apoptosis observed in the *cisd-1(tm4993)* mutant and *cisd-1(RNAi)* animal is through the core apoptotic machinery. Using the ACT-5::YFP or CED-1::GFP programmed cell death reporters, we determined that *ced-3* function is required for the increase in programmed cell death observed in animals with *cisd-1* dysfunction (Fig. 6, Figure S5, Figure S6). The *cisd-1(tm4993); ced-3(RNAi)* animal had a significantly reduced number of cell corpses relative to that observed in the *cisd-1(tm4993)* (Figs. 6a, b). Similar results were observed in the *cisd-1(RNAi); ced-3(RNAi)* animal using the ACT-5::YFP (Figure S5a, b) or in the *cisd-1(tm4993)*; *ced-3(RNAi)* using the CED-1::GFP reporter strain (Figure S6a, b). Furthermore, there was a significant decrease in the number of cell corpses in the *cisd-1(RNAi); ced-4(n1162)* animal relative to the *cisd-1(tm4993)* mutant (Figure S6a, c). Combined, these results indicate that the increase in cell corpses observed in the *cisd-1(tm4993)* mutant or *cisd-1(RNAi)* animal required the activity of CED-3 and CED-4.

During our analysis, we noticed that the knock-down of *ced-3* function by RNAi did not suppress the Mig phenotype within the germline of the *cisd-1(tm4993)* animal (Figure S7). Others have shown that *ced-5* or *ced-10* dysfunction results in a Mig phenotype [31]. The knock-down of *ced-5* or *ced-10* via RNAi in the *cisd-1(tm4993)* mutant did not further increase the Mig phenotype relative to the single mutant (Figure S7). Together, these results suggest that the increased cell corpses within the germline and the germline Mig phenotype are a result of distinct dysfunctions in the *cisd-1(tm4993)* animal.

The CED-9 protein functions to prevent cells from dying. Thus, the *ced-9(n1950gf)* gain-of-function mutant has been instrumental in examining the prevention of cell deaths within the embryo [38] or germline [20, 24]. Using the ACT-5::YFP reporter, we tested if the *ced-9(n1950gf)* allele would reduce the number germline cell death in the *cisd-1(tm4993)* animals. The *cisd-1(tm4993); ced-9(n1950gf)* animal had a significantly reduced number of cell corpses relative to *cisd-1(tm4993)* animals (Figs. 6a, c). The number of cell corpses was not completely eliminated in the *ced-9(n1950gf); cisd-1(tm4993*) mutant, which is in line with the previous reports that physiological germline apoptosis is not completely prevented by the *ced-9(n1950gf)* mutation [20, 24]. These data provide further evidence that the increased cell death observed in the *cisd-1(tm4993)* animals is due to altered regulation of the core apoptotic machinery.

**Fig. 7:**
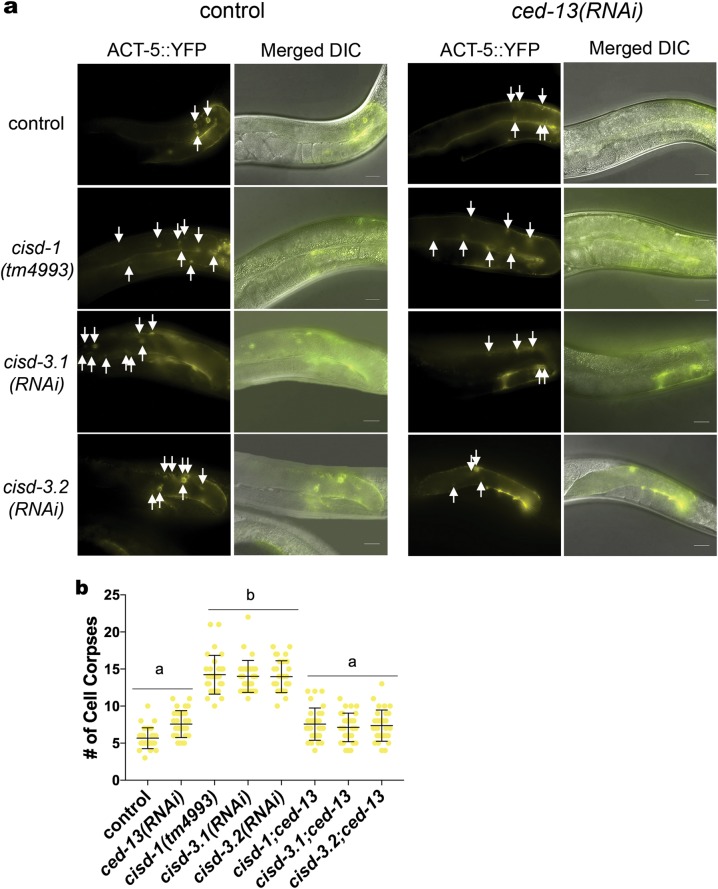
The number of cell corpses within the gonad is reduced in the *cisd* animals with disrupted *ced-13* function. Using the ACT-5::YFP reporter strain to visualize apoptotic cells, the number of apoptotic cells was quantified in the *cisd-1(tm4993), cisd-3.1(RNAi)* *and cisd-3.2(RNAi)* animals with *ced-13* knock-down. **a** Representative images of the gonad arm of control, *cisd-1(tm4993), cisd-3.1(RNAi)*, and *cisd-3.2(RNAi)* animals with or without knock-down of *ced-13* by RNAi. The ACT-5::YFP reporter is shown individually and merged with the DIC image. White arrows point to apoptotic cells within the gonad. Scale bar = 20 µm. **b** Disruption of *ced-13* function by RNAi reduced the number of cell death corpses within the gonad of the *cisd-1(tm4993), cisd-3.1(RNAi), cisd-3.2(RNAi)* animals. Identical letters indicate groups with no significant differences; different letters indicate *P* < 0.0001 (Kruskal–Wallis, Dunn’s multiple comparison test). The number of apoptotic corpses within the gonad were quantified in animals from three independent experiments for a total of at least thirty animals. Error bar represents standard deviation.

In *C. elegans*, the *egl-1* and *ced-13* genes code for two pro-apoptotic, BH3 (Bcl-2 homology region 3) proteins that physically interact with and regulate the anti-apoptotic CED-9 protein [39–41]. Using the ACT-5::YFP reporter strain, we tested if *ced-13(RNAi)* or *egl-1(RNAi)* reduced the number of apoptotic cells within the germline of *cisd-1(tm4993)* animals. The number of cell death corpses within the germline was not significantly different in the *cisd-1(tm4993); egl-1(RNAi)* animal relative to the *cisd-1(tm4993)* animal (Figure S8). This is consistent with the idea that loss-of-function *egl-1* does not impact physiological germline cell death [24]. However, the *cisd-1(tm4993); ced-13(RNAi)* animal had a significant reduction in cell death corpses relative to the *cisd-1(tm4993)* animal (Fig. 7). Furthermore, *ced-13(RNAi)* significantly reduced the number of cell death corpses within the germline of *cisd-3.1(RNAi)* and *cisd-3.2(RNAi)* animals (Fig. 7). To confirm that *ced-13* dysfunction suppressed the increased programmed cell death observed within the *cisd-1(tm4993)* germline, we crossed in the *ced-13(sv32)* loss-of-function allele into the *cisd-1(tm4993)* mutant. The *cisd-1(tm4993)*; *ced-13(sv32)* double mutant had a significant reduction in cell death corpses within the germline relative to *cisd-1(tm4993)* animal (Figure S9). Combined, these data demonstrate that the *cisd* gene family could function as regulators of physiological germline programmed cell death acting through *ced-13* function.

## Discussion

Disruption of the *cisd* gene family in *C. elegans* resulted in various germline defects including increased physiological apoptosis and DTC migration defects. A previous study examining the phenotypes associated with disrupted *cisd* gene function in mammalian cells also noted an increase in apoptosis as determined by biochemical assays, histochemistry and annexin staining, but did not determine whether this activation directly involved core programmed cell death members such as BCL-2 [30]. Here we demonstrate that the CISD family members play a pro-survival role in the process of apoptosis and that they function through the Bcl-2 family CED-9 and BH3 domain CED-13 but not through EGL-1. Furthermore, we show that the CISD-3 proteins in *C. elegans* have overlapping functions with CISD-1.

Given that the knock-down of any one of the *cisd* gene family members increased germline apoptosis, we propose that they have overlapping functions in *C. elegans*. Furthermore, we suggest that the CISD proteins work in a cooperative manner and that disruption of one member of the gene family alters that cooperativity. This idea is in line with the findings that NAF-1/CISD2 and mitoNEET/CISD1 physically interact and cooperate in their function in mammalian cells [42]. Given that *ced-3(RNAi), ced-4(n1162)* and *ced-9(n1950gf)* suppressed the increased germline programmed cell death observed in *cisd-1(tm4993)* and/or *cisd-1(RNAi)* animals, we propose that CISD proteins function upstream of the core apoptotic machinery.

**Fig. 8:**
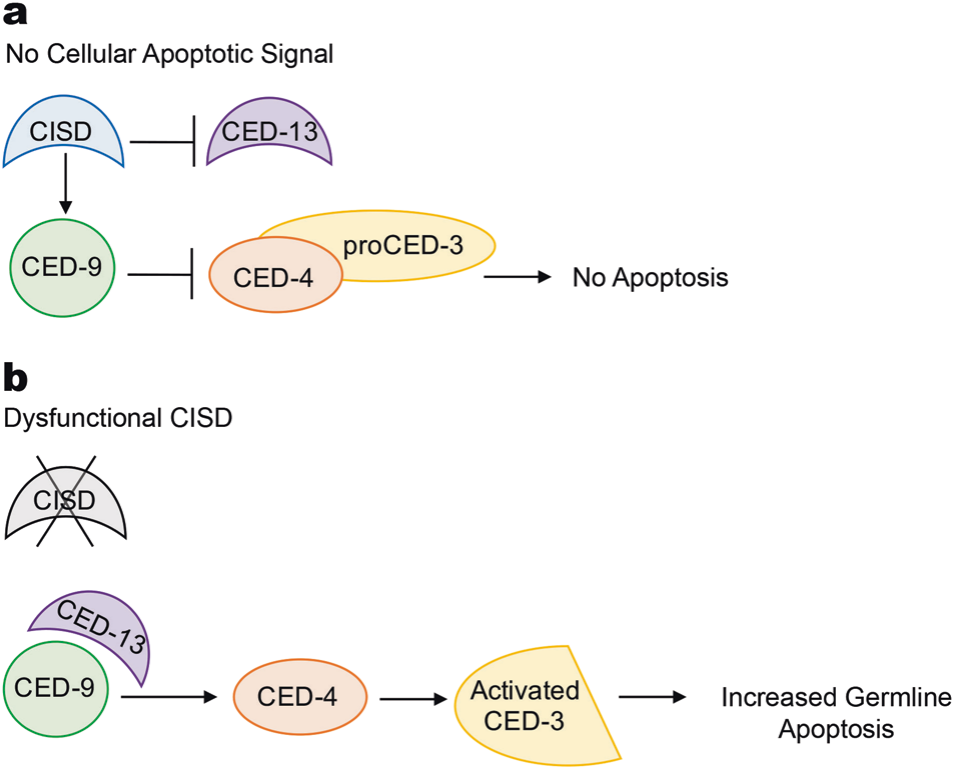
Model of CISD function. We propose that the CISD protein family and CED-13 compete to regulate CED-9 function in the process of physiological germline apoptosis. **a** In the absence of a cellular signal that initiates physiological germline apoptosis, CISD functions in an anti-apoptotic/pro-survival manner. However, it is not known if this is by CISD protein(s) interacting with CED-9 and/or CED-13. **b** The dysregulation of any member of the *cisd* gene family results in increased germline apoptosis. This could be through increased CED-13 inhibition of pro-survival CED-9, given that *ced-13(RNAi)* suppress the increased germline apoptosis observed in animals with disrupted *cisd* function.

Figure 8 communicates our working model of how the CISD family functions within the physiological germline apoptosis pathway. This proposed model is based on our results presented here, the findings in the mammalian system that the NAF-1/CISD2 protein interacts with Bcl-2 at the same domain that the CED-13-related protein Bik binds [2], and that *C. elegans* CED-13 and CED-9 physically interact [40]. This model is further supported by research showing that peptides specific for the BH3 domains of CED-13 or EGL-1 interact with CED-9 and that this interaction disassociates CED-9 from CED-4; presumably this would initiate apoptosis in vivo [43]. We propose that the CISD proteins act in a pro-survival/anti-apoptotic manner, whereas CED-13 acts in a pro-apoptotic manner. CISD and CED-9 interaction could promote germ cell survival by inhibiting CED-9/CED-13 interaction (Fig. 8a). When the CISD function is disrupted, or in response to a signal initiating physiological cell death in the germline, there is an increase in CED-9/CED-13 interaction, which will in turn release CED-4 to activate CED-3 function (Fig. 8b). This model is in line with our finding that *ced-13(RNAi)* reduces the increased programmed cell death observed in the animals with disrupted *cisd* function. This is also consistent with previous studies in mammalian cell culture that show that NAF-1 binds to Bcl-2 at the same site in which BH3 domain proteins bind [1, 44].

We showed that *cisd* dysfunction resulted in various germline abnormalities, including DTC migration defects. The *cisd-1(tm4993); ced-3(RNAi)* animals continued to display the Mig phenotype in the absence of germline apoptosis. Thus, the DTC migration defect and increased germline apoptosis are likely due to alterations of two independent processes. Furthermore, it is likely that the *cisd* gene family in *C. elegans* is involved with various cellular processes. Other genes that cause a Mig phenotype when dysfunctional are involved with cell adhesion, pathfinding and cell death engulfment genes [31, 45]. It will be of interest to examine these non-apoptotic CISD functions in the context of pathways regulating cell migration.

As the pioneering work conducted in *C. elegans* to identify the molecular mechanisms regulating apoptosis within the embryo, subsequent studies have provided insights into germline programmed cell death [24, 26, 39, 46]. Although much work has been conducted, there remain questions regarding the regulation of physiological germline apoptosis. For example, it is not completely clear what triggers physiological apoptosis or why some germ cell fates undergo apoptosis and others do not. It will be of interest to examine these questions in the context of CISD function.

## Materials and methods

### Strains and culture conditions

Worms were raised and maintained at 20 °C on NGM (nematode growth media) and seeded with *Escherichia coli*(*E. coli*) (OP50) bacteria for food source, unless otherwise noted [47]. The following strains were acquired from the *Caenorhabditis* Genetics Center (CGC): N2, CU1546 [*ced-1p::ced-1::GFP* *+* *rol-6(su1006)*], WS2170 [*lim-1*p::YFP::*act-5* + *unc-119(ed3)*], KX89 [*ced-4(n1162); lim-7p::ced-1::GFP* *+* *lin-15*] [48], MT4770 [*ced-9(n1950gf)*], MT3608 [*unc-13(ed42)*; *ced-1(e1735)*] and MD792 [*ced-13(sv32)*]. The *ced-9(n1920)* gain-of-function mutation is a point mutation affecting the *ced-9* open reading frame resulting in a glycine to glutamate substitution [25]. The *ced-4(n1162)* allele contains a premature stop codon [49]. The MD792 strain [*ced-13(sv32)*] contains a deletion that removes the entire *ced-13* coding region and 747-bp upstream of the ATG codon and 209-bp downstream of the TAA stop codon [40]. The *cisd-1(tm4993)* mutant was obtained from the National BioResource Project (NBRP) [50] and back-crossed three times to N2 wild-type animals to produce strain PM147. The *cisd-1(tm4993)* mutant contains a 316-bp deletion that removes part of the coding region of both *cisd-1a* and *cisd-1b* (Figure S1b). The deletion removes a portion of intron 1 through exon 3 for *cisd-1b* and a portion of the 5′-UTR to exon 2 for *cisd-1a*. The deletion does not remove any coding region of adjacent genes. The PM147 strain was used to produce the following strains via genetically crossing with strains described above: PM151 [*cisd-1(tm4993); ced-1p::ced-1::GFP* *+* *rol-6(su1006)*], PM149 [*cisd-1(tm4993); lim-1*p::YFP::actin + *unc-119(ed3)]*, PM153 [*cisd-1(tm4993); ced-9(n1950gf); lim-1*p::YFP::actin + *unc-119(ed3)*] and PM162 [*lim-1*p::YFP::actin + *unc-119(ed3); ced-13(sv32); cisd-1(tm4993)*]. The MT4770 [*ced-9(n1950gf)*] strain was used to produce strain PM152 [*ced-9(n1950gf); lim-1*p::YFP::actin + *unc-119(ed3)*]. The MD792 strain (*ced-13(sv32)*) was used to produce the PM161 strain [*lim-1*p::YFP::actin + *unc-119(ed3); ced-13(sv32)*]. The PM154 strain [*cisd-1(pnls27*[GFP^SEC^3xFlag::*cisd-1*])] was generated using the CRISPR technique as outlined below. The genotypes of strains produced by genetic crosses were verified by PCR (Table S1). DNA sequencing was also used to verify the genotypes of strains produced by crossing in the *ced-9(n1950gf)* allele.

### CISD protein family sequence alignment

To examine sequence homology of the CISD proteins, multiple sequence alignments of the CISD protein sequences (FASTA format, *Homo sapiens* and *C. elegans)*, were conducted using the Clustal Omega (1.2.4) web interface program [51]. The output format parameters were set for Clustal alignment without numbers. The CISD-1 isoforms were aligned with the mitoNEET/CISD1 and Naf-1/CISD2 human protein sequences. The *C. elegans* CISD-3.1 and CISD-3.2 protein amino-acid sequences were aligned with the human Miner2/CISD3 protein sequence.

### Generation of the cisd-1(pnls27) insertion and GFP reporter strain

We used the CRISPR/Cas9-based approach developed by Dickenson et al. to produce a GFP transcriptional reporter and insertion strain [29]. The *cisd-1* gene is predicted to code for two isoforms; *cisd-1a* contains two exons and *cisd-1b* contains three exons (Fig. 3a, Figure S1b). To disrupt both isoforms and produce a transcriptional GFP reporter for *cisd-1*, the CRISPR construct was designed such that the GFP insertion interrupted the first exon of *cisd-1a* and the second exon of *cisd-1b*. The Zhang’s lab CRISPR design tool website (crispr.mit.edu) was used to determine Cas9 target sequence. The protocol was designed for GFP^SEC^3xFlag insertion at 536-bp downstream of the start site. The pDD162 vector (Addgene plasmid #47549) was used to produce the *cisd-1* Cas9-sg RNA (*pCas9-sgc; cisd-1*). The FP-SEC vector pDD282 (Addgene plasmid #66823) was used to produce the repair template plasmid for homologous recombination at the *cisd-1* locus (*GFP^SEC^3XFlag::cisd-1*). The NEB Q5 Site-Directed Mutagenesis Enzyme Kit was used to produce the plasmid constructs. The approach for producing plasmid and microinjection mixtures were from the Goldstein Lab Self Excising Cassette Protocol [29]. Synchronized 1-day old adult hermaphrodites were obtained and the DNA solution was micro-injected into the proximal gonad using an inverted compound microscope [52]. After injection, worms were recovered and placed onto individual OP50 seeded plates and nematode candidates containing the GFP^SEC^3xFlag insertion were selected for by animals exhibiting hygromycin resistance and a roller phenotype [29]. Three candidates were isolated; the PM154 strain, [*cisd-1(pnls27* [GFP^SEC^3xFlag::*cisd-1*]) II], produced the most progeny with the roller phenotype and thus was used for analysis. The generated lines were verified by genotyping using PCR and DNA sequencing; the primer sequences used for this experiment are included in Table S1.

### RNAi assays

RNAi was conducted as previously described [53, 54]. The HT115 and RNAi *E. coli* strains were obtained from the Medical Research Council Geneservice, Source BioSciences (Cambridge, UK) or produced by our lab as outlined below [45, 55]. We produced RNAi strains, using standard DNA cloning techniques, to target *cisd-1* or *cisd-3.2* as these strains are not present in the Ahringer library. To construct the *cisd-1* RNAi bacterial strain, 367 bp of the *cisd-1* gene, which contained exons 2 and 3 and intron 2, was cloned into the L4440 vector to produce the plasmid pL440cW02B12.15. To construct the *cisd-3.2* RNAi bacterial strain, 1564 bp of the *cisd-3.2* gene, which encodes exons 1, 2 and 3 and introns 2 and 3, was cloned into the L4440 vector to produce plasmid pL440cY67D2.3. These DNA sequences were cloned so that the T7 promoter could generate the double-stranded RNA in the presence of IPTG (isopropyl-β-D-1-thiogalactopyranoside). The DNA within the plasmids were verified by DNA sequencing. The plasmids were transformed into HT115(DE3) bacteria. To conduct the RNAi experiments, animals were raised on NGM plates (0.5 g/ml IPTG, 200μg/ml ampicillin, 12.5μg/ml tetracycline) seeded with either HT115 control food (L4440 plasmid with no insert) or the *E. coli* strain for RNAi of the specified gene. Briefly, embryos were grown to 1-day old adulthood and the F1 embryos were transferred to fresh NGM IPTG plates seeded with the appropriate bacteria. The F1 embryos were raised on the RNAi food and analyzed at the developmental stage indicated for each experiment. The RNAi food to target *par-6* was used as an internal control to test reagents effectiveness, because *par-6(RNAi)* leads to obvious phenotypes within the P0 and F1 generation (sterility, embryo lethality).

### Quantitative RT-PCR

One-day old adult hermaphrodites were collected for mRNA isolation and RT-PCR as previously described [56, 57]. Briefly, mRNA was isolated using TRIzol Reagent (Life Technology, 15596026), NucleoSpin RNA Clean-up (Machenerey-Nagel, 740948-50) and TURBO DNA-free^TM^ kit (Life Technology, AM1907) reagents. The complementary DNA was generated using the SuperScript III synthesis system (Invitrogen, 18080-051). The quantitative RT-PCR was carried out using a StepOnePlus real-time PCR system (Applied Biosystems) and Fast SYBR Green Master Mix (Applied Biosystems, 4385612). The mRNA level of Y45F10D.4 was used for normalization [58]. The average of three technical replicates was used for each experiment and the three independent biological replicates were statistical analyzed using one-way analysis of variance (ANOVA).

### Germline phenotype analysis

The number of progeny produced by *cisd-1(tm4993)* and *cisd-1(pnIs27)* mutants, relative to N2 control, was determined as previously described [57]. At least four synchronized animals were collected at the L4-to-adult molt, placed as individuals onto a NGM plate and allowed to lay eggs over a 24-h period. The adult worms were moved every 24 h and the progeny produced during each 24-h interval were counted after hatching. Animals were examined until no progeny were produced. To examine the anatomy and morphology of the hermaphrodite gonad, DIC microscopy was used to visualize the gonad within L4 larvae and 1-day old adults; only gonads that were completely visible were scored [24, 34]. Briefly, the proximal gonad arm was examined for the number of maturing oocytes by counting the oocytes from the primary oocyte to the region in the germline where the germ cells are in a non-linear arrangement (near the turn). The animals were scored as having a distal tip cell migration (Mig) defect if the morphology of the gonad was abnormal due to extra turns or abnormal turning was observed. For each experiment, at least three independent experiments were completed.

### Programmed cell death assays

To quantify the germ cell corpses, synchronized L4 larvae were collected and the germline was examined 48 h later. The cell corpses within the germline were counted using DIC microscopy or fluorescent microscopy to visualize reporter strains or acridine orange staining [24, 34]. Using DIC microscopy, the number of cell corpses quantified include early stage apoptotic cells (corpses begin to cellularize) and the middle to late stage of apoptosis (corpses appear as “buttons” after cellularization out of the germline syncytium) [34]. The cell corpse reporter strains used include the CU1546 strain [*ced-1p::ced-1::GFP* *+* *rol-6(su1006)*] and KX89 strain [*ced-4(n1162); lim-7::ced-1::GFP* *+* *lin-15*] to quantify the number of cells going through the process of engulfment [36], and the WS2170 strain [*lim-1*p::YFP::*act-5* + *unc-119(ed3)*], to quantify the early stages of apoptosis [35]. The acridine orange (Sigma) staining protocol was conducted as previously described [24] with the following modification; the adult hermaphrodites were incubated on NGM plates, seeded with OP50 and 500 µl of acridine orange dissolved in M9 for a final concentration of 0.02 mg/ml; animals were placed in the dark for 2 h prior to visualization. To minimize the acridine orange within the intestine, which can interfere with germline visualization, the animals were transferred to OP50 seeded NGM plates for 1 h before analysis.

DIC microscopy analysis of N2 wild-type, *cisd-1(tm4993)* and *ced-1(e1735)* L1 larvae, within 1.5 h of hatching, was conducted to quantify the number of persistent cell corpses as previously described [59]. A total of 30 animals from three independent trials were examined. DIC microscopy analysis of N2 wild-type, *cisd-1(tm4993)* and *ced-1(e1735)* bean or comma stage embryos was conducted to quantify the number of somatic programmed cell death corpses. The dying cells, which appeared as refractile discs that are morphologically distinct from other cells, were quantified as previously described [59].

### Nomarski microscopy analysis

Microscopy analysis was conducted similarly as previously described [60]. Briefly, nematodes were mounted on a 3% agarose slide containing an anesthetic solution (0.1% tricane, 0.01% levamisole). Animals were examined using a motorized Zeiss Axioscope fluorescent microscope. Images were collected using the AxioVision 4.7.1 software (Zeiss) and processed using ImageJ (NIH) and Adobe Photoshop CC (Adobe Systems Inc.) software.

### Statistics

All data sets are expressed as mean ± standard deviation (SD). Data sets were analyzed for Gaussian distribution using D’Agostino–Pearson Omnibus normality test or Shapiro–Wilk normality test (alpha = 0.05, *P* > 0.05). If a normality test was passed, a parametric statistical test was performed; if a normality test was not passed then the data were analyzed using a nonparametric statistical test. Statistical tests conducted include: unpaired *t-*test, ordinary one-way ANOVA followed by a Dunnett’s multiple comparison test, Kruskal–Wallis test followed by a Dunn’s multiple comparisons test or two-way ANOVA followed by a Sidak’s multiple comparisons test. The statistical test for each experiment is as indicated in the figure legends. *P*-values are reported with each data set. Data were graphed and analyzed using GraphPad Prism 7.0b.

## Electronic supplementary material

Figure S1

Figure S2

Figure S3

Figure S4

Figure S5

Figure S6

Figure S7

Figure S8

Figure S9

Table Sq

Supplementary figure legends
