# Supplementary figures and images for: The cisd gene family regulates physiological germline apoptosis through ced-13 and the canonical cell death pathway in Caenorhabditis elegans
Source: Cell Death Differ. 2018 Apr 17;26(1):162–78. doi: 10.1038/s41418-018-0108-5 (PMC6294797; doi:10.1038/s41418-018-0108-5)

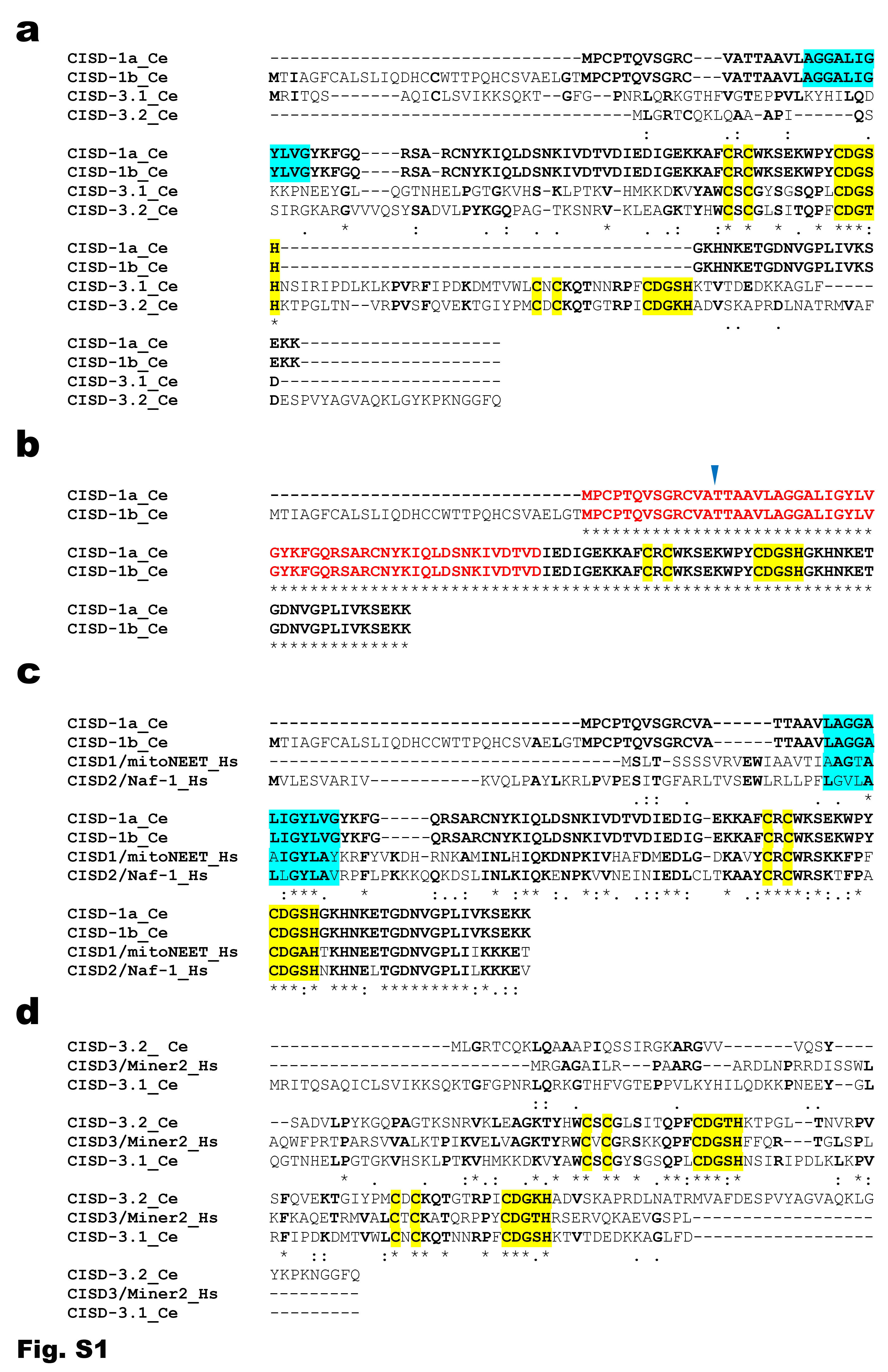

Supplement: Supplementary file 1 — Figure S1 [file 41418_2018_108_MOESM1_ESM.jpg]

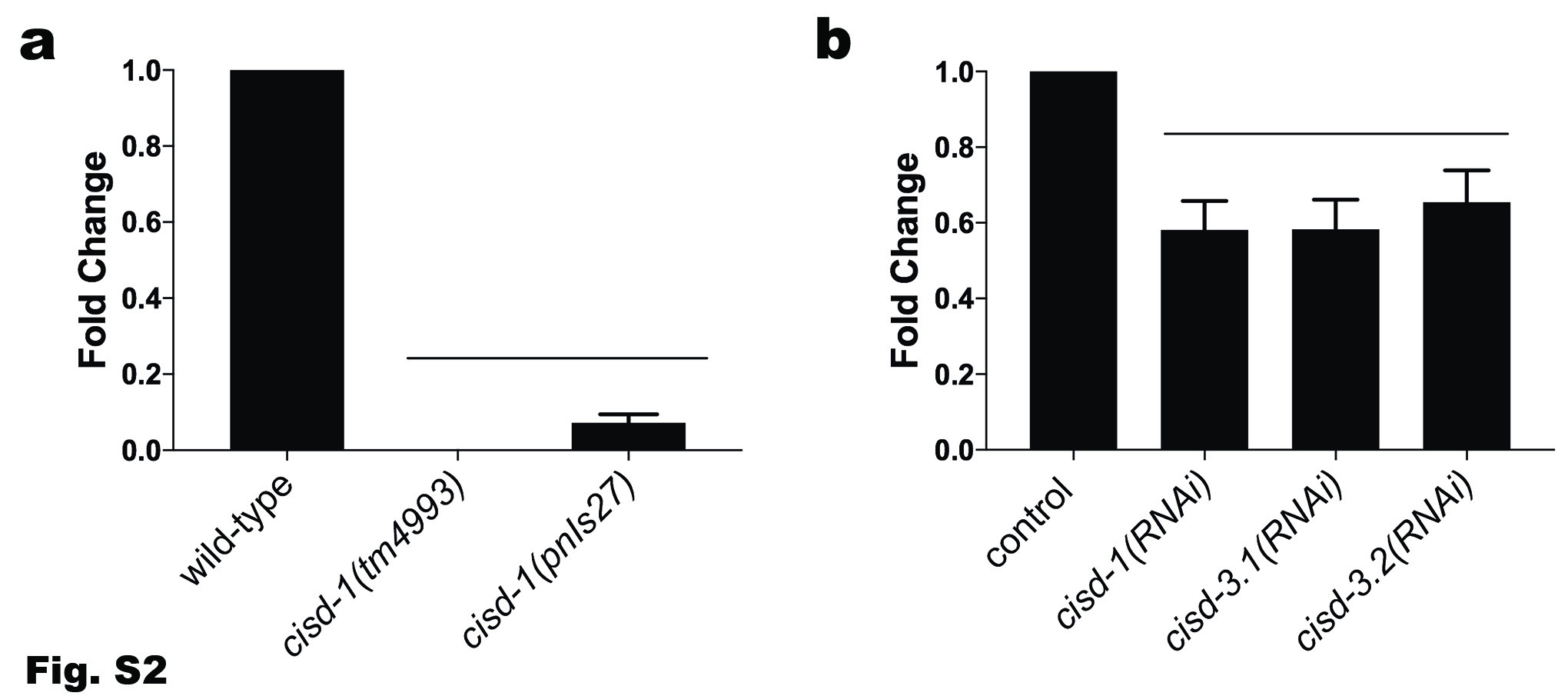

Supplement: Supplementary file 2 — Figure S2 [file 41418_2018_108_MOESM2_ESM.jpg]

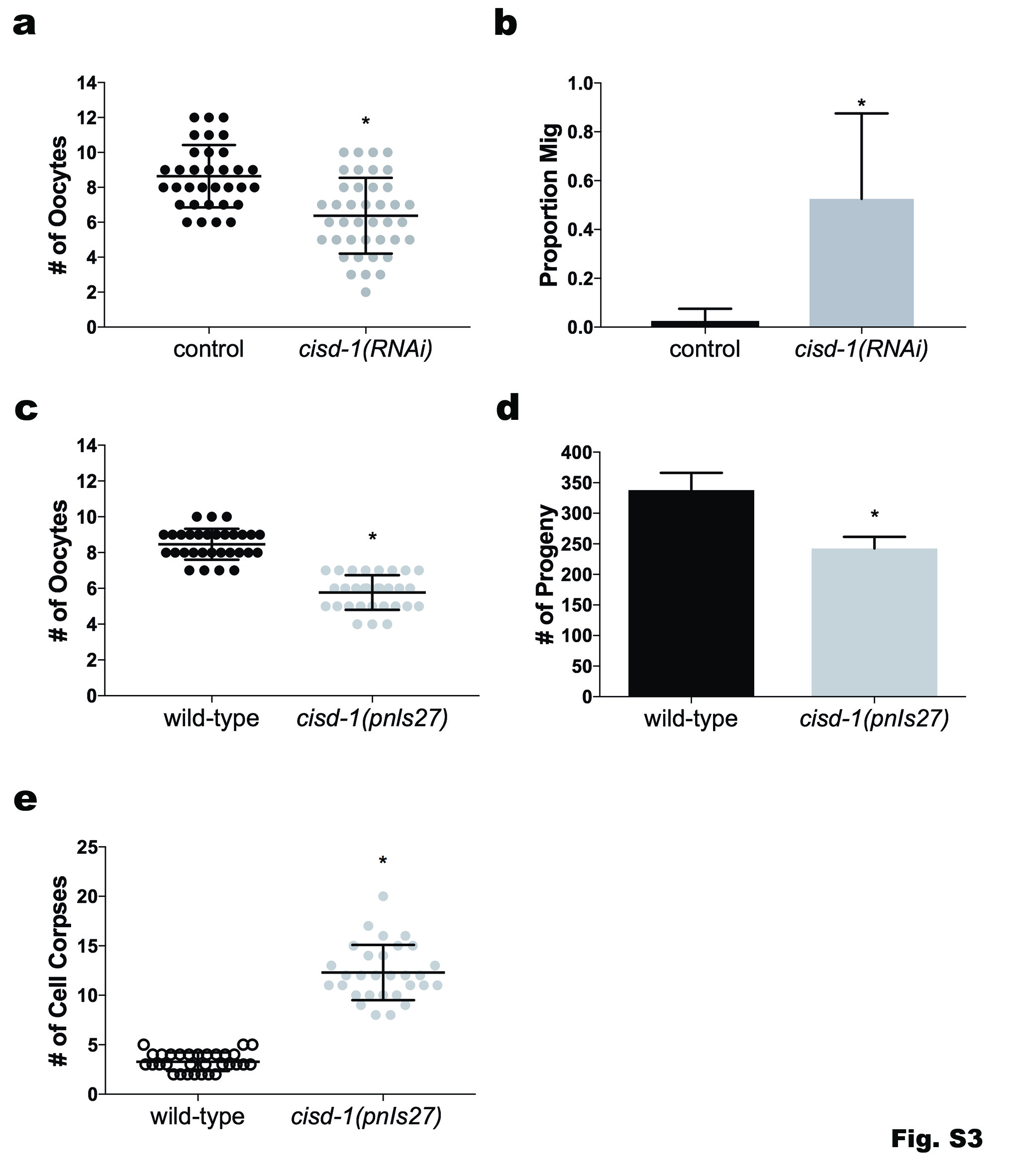

Supplement: Supplementary file 3 — Figure S3 [file 41418_2018_108_MOESM3_ESM.jpg]

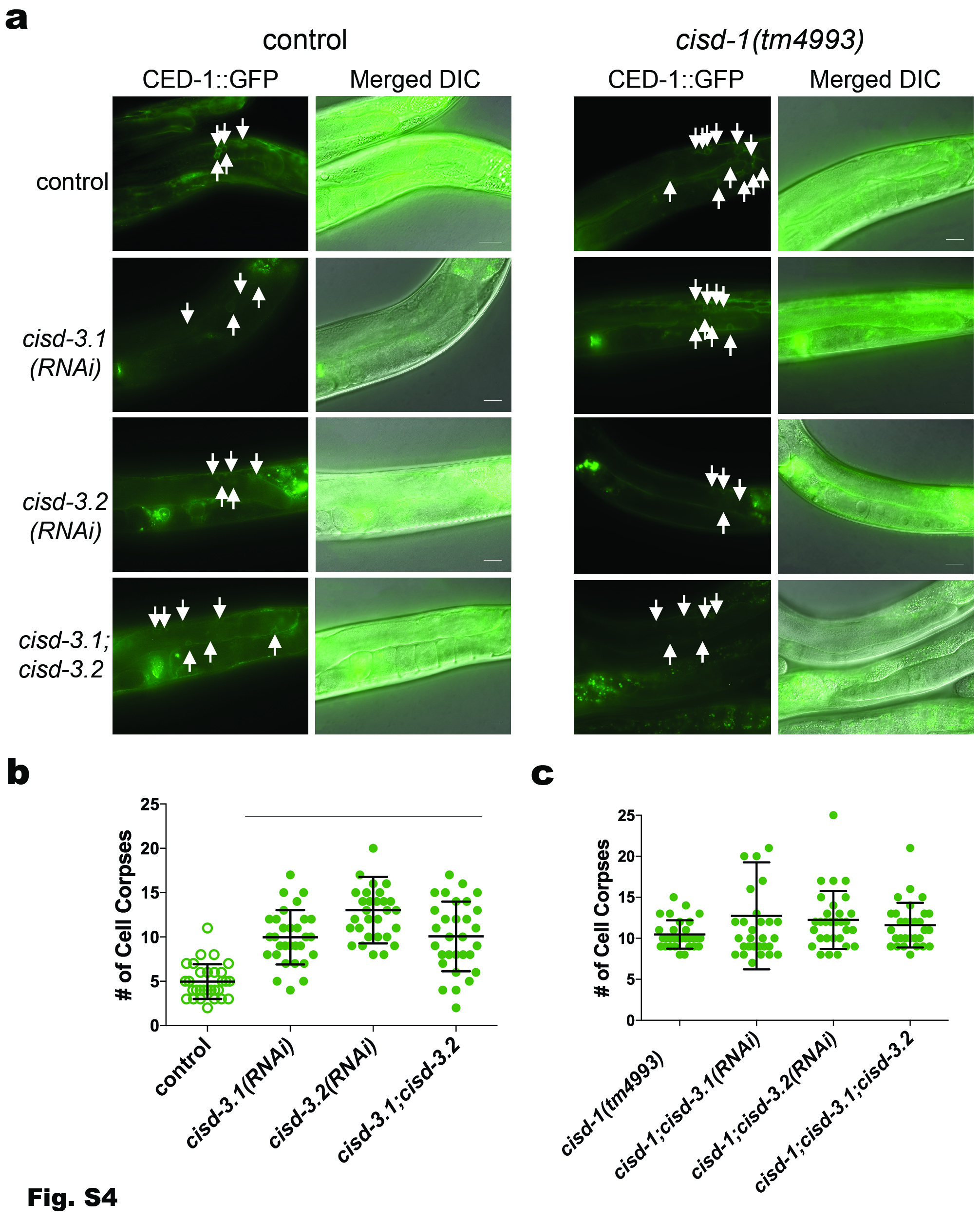

Supplement: Supplementary file 4 — Figure S4 [file 41418_2018_108_MOESM4_ESM.jpg]

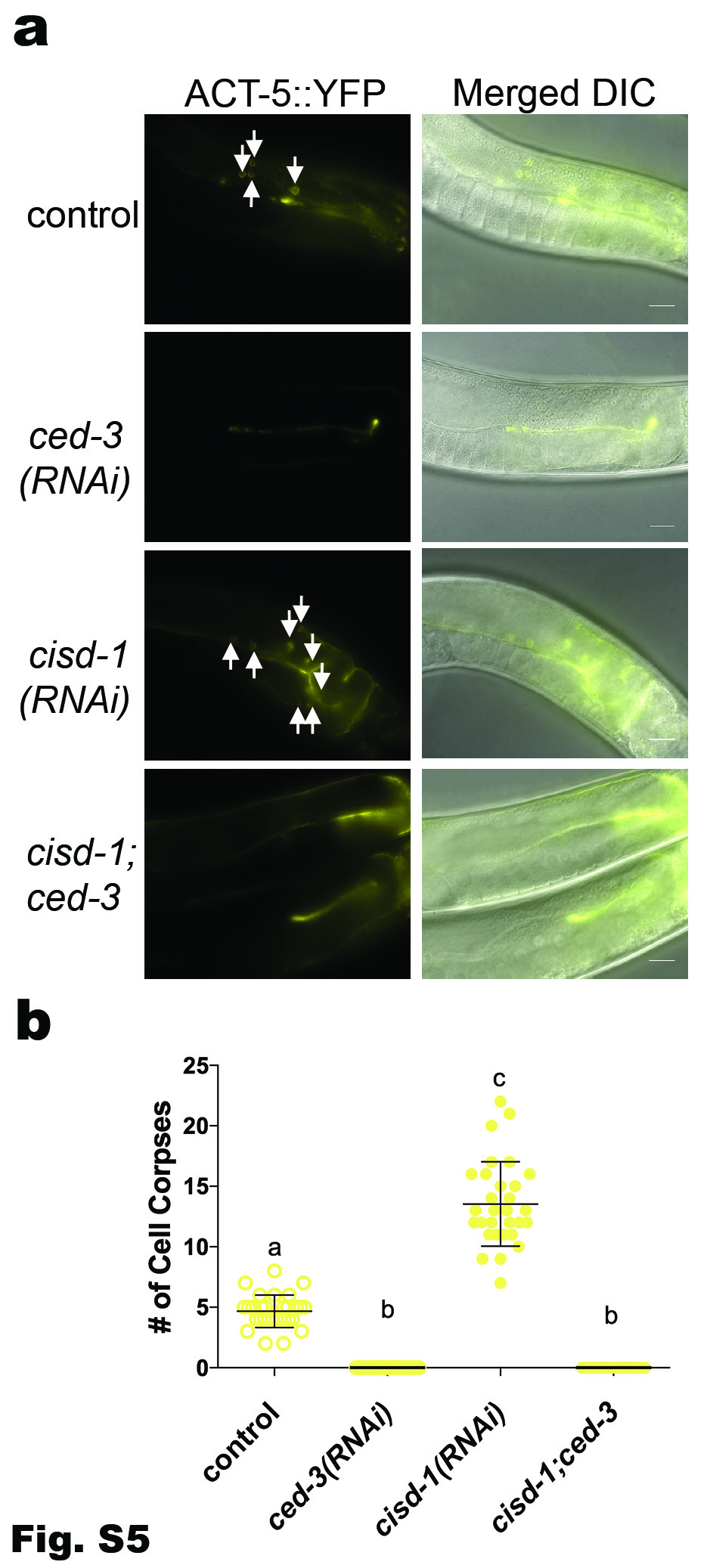

Supplement: Supplementary file 5 — Figure S5 [file 41418_2018_108_MOESM5_ESM.jpg]

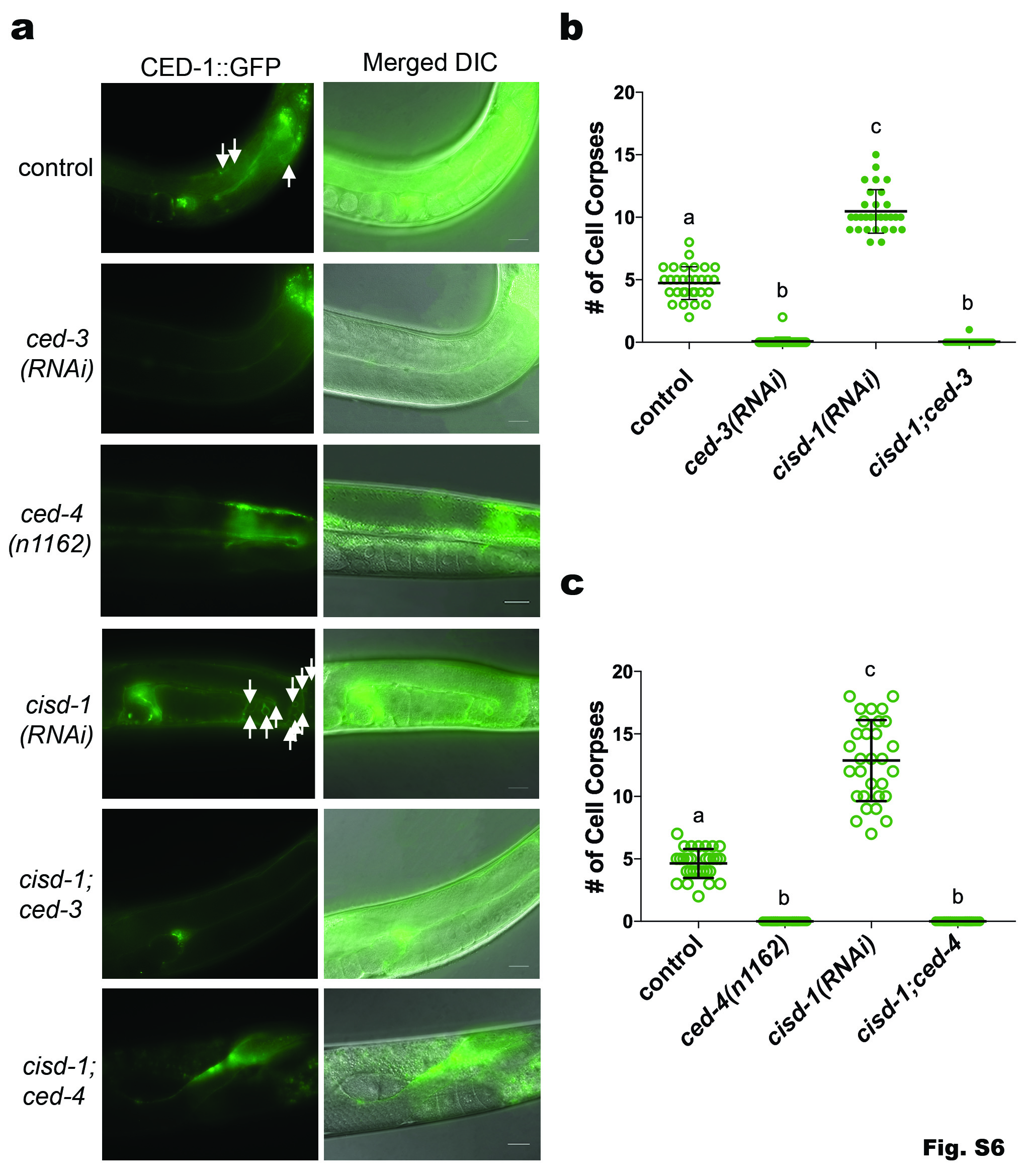

Supplement: Supplementary file 6 — Figure S6 [file 41418_2018_108_MOESM6_ESM.jpg]

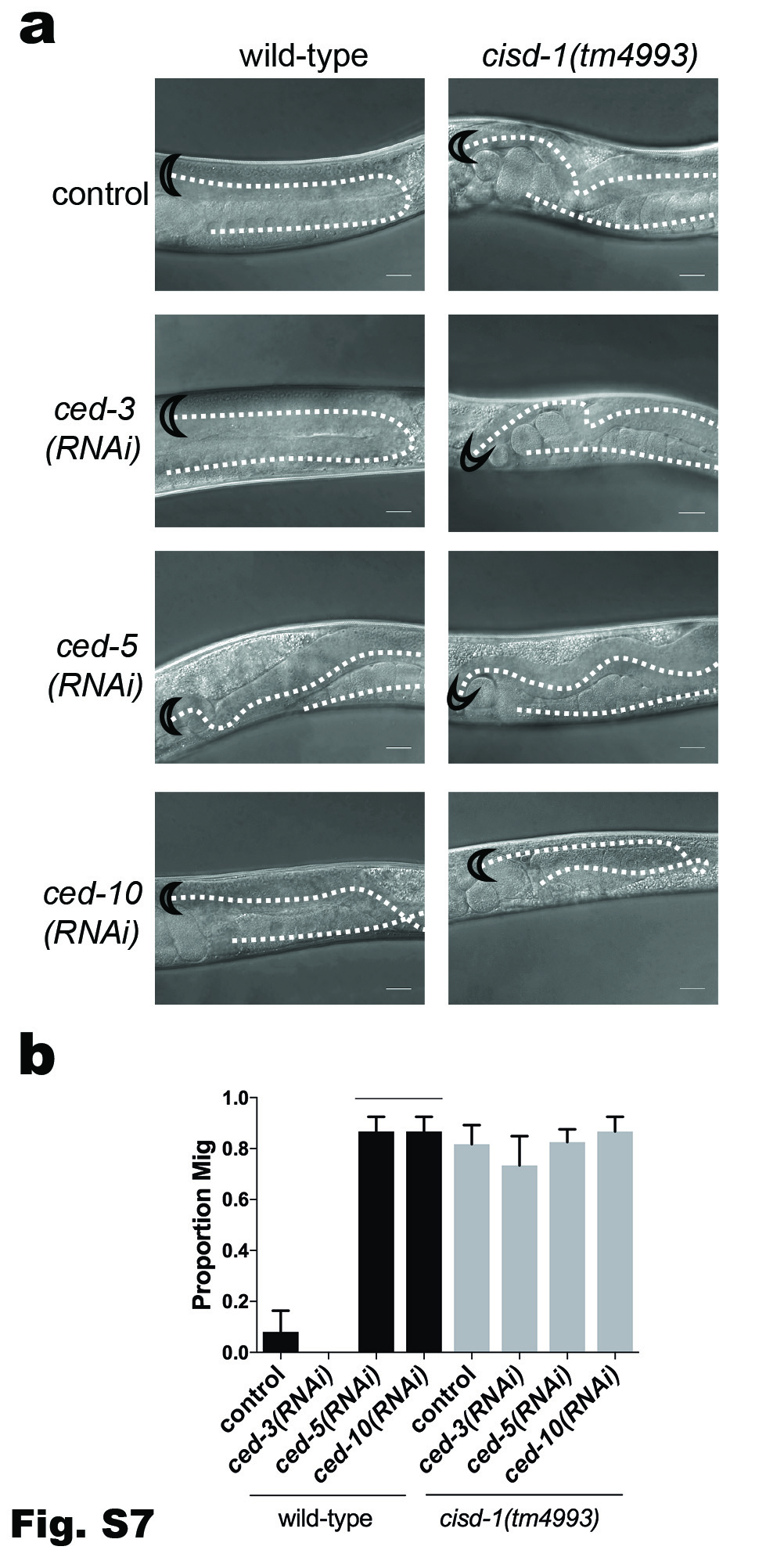

Supplement: Supplementary file 7 — Figure S7 [file 41418_2018_108_MOESM7_ESM.jpg]

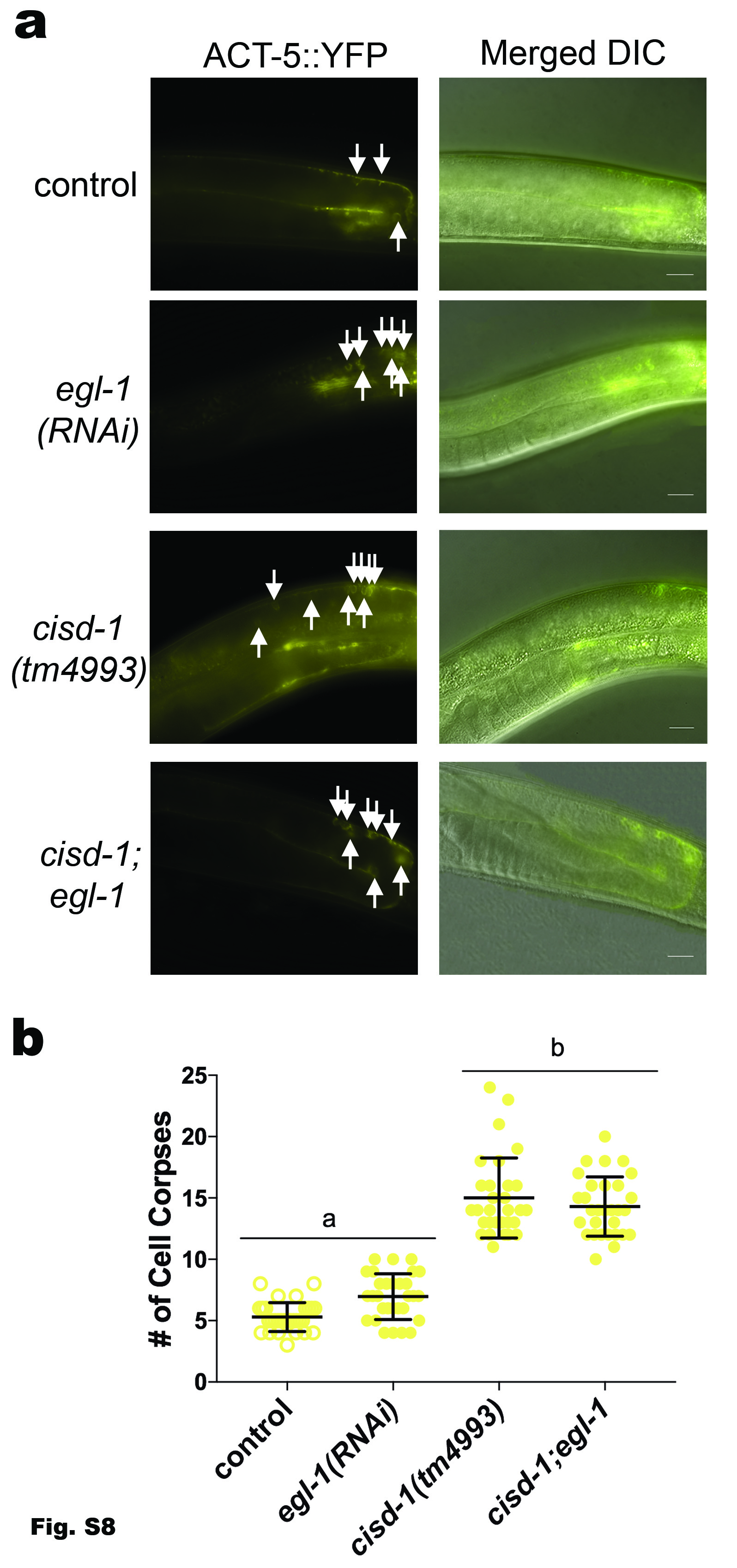

Supplement: Supplementary file 8 — Figure S8 [file 41418_2018_108_MOESM8_ESM.jpg]

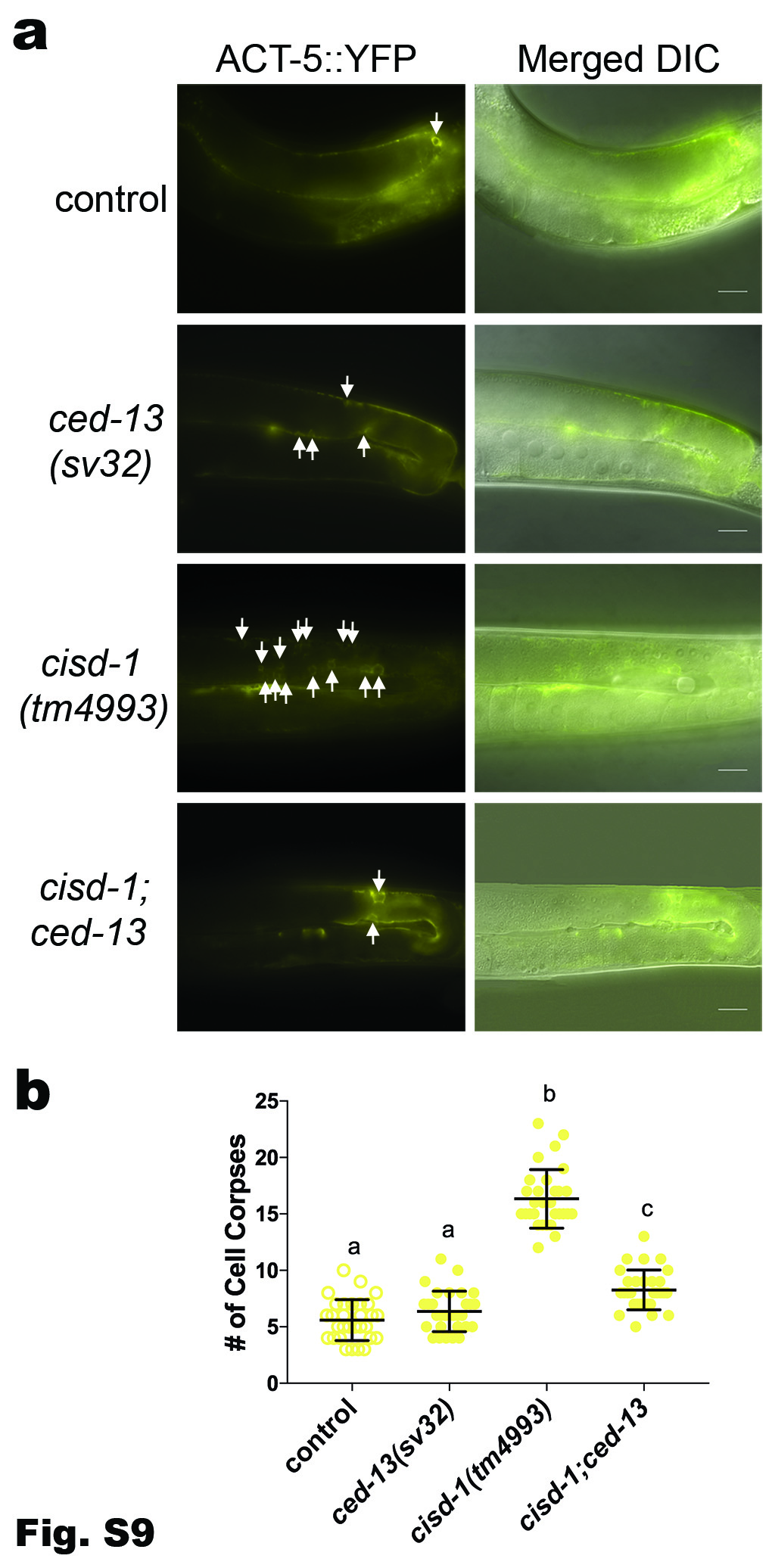

Supplement: Supplementary file 9 — Figure S9 [file 41418_2018_108_MOESM9_ESM.jpg]
