# Supplementary material for: The cisd gene family regulates physiological germline apoptosis through ced-13 and the canonical cell death pathway in Caenorhabditis elegans
Source: Cell Death Differ. 2018 Apr 17;26(1):162–78. doi: 10.1038/s41418-018-0108-5 (PMC6294797; doi:10.1038/s41418-018-0108-5)
Supplement: Supplementary file 10 — Table Sq [file 41418_2018_108_MOESM10_ESM.pdf]

Table S1. Primers used in study

Gene (Allele)	Primer Use	Forward Sequence	Reverse Sequence
cisd-1(tm4993)	genotyping	tatggccacttctcgctctt	tgttctgttgcagagcttgg
ced-9 wildtype	genotyping	caatgtccaatgtcttatag	tcgaattcgtaaagttttctg
ced-9(n1950gf)	genotyping	caatgtccaatgtcttatca	tcgaattcgtaaagttttctg
ced-9*(n1950gf)*	sequencing	atgacacgctgcacggc	tcgaattcgtaaagttttctg
ced-13(sv32)	genotyping	ccagctgtagtgtttcatgt	acgtggatagccctgatacc
cisd-1(RNAi)	plasmid construct	gagaagatctatgccttgcccaactcaa	gagactcgagttacttcttttcggatttg
cisd-3.2(RNAi)	plasmid construct	gagaagatctatgctcggaagaacgtgc	gagactcgagatcagcgtgttttccatc
cisd-1(pnls27)	Cas9-sgRNA target	acgactgccgccgttctcgcgttttagagctag aaatagcaagt	caagacatctcgcaatagg
cisd-1(pnls27)	Left Homology Arm	acgttgtaaaacgacggccagtcgccggcatt ttcttcttctcacgtccaaat	catcgatgctcctgaggctcccgatgctccggca gtcgtggcgacacagcgac
cisd-1(pnls27)	Right Homology Arm	cgtgattacaaggatgacgatgacaagagat aagagctcttattggataccttgtaag	ggaaacagctatgaccatgttatcgatttctcggtc caaatttgacgtggccaac
Y45F10D.4	RT-PCR	gtcgcttcaaatcagttcagc	gttcttgtcaagtgatccgaca
cisd-1	RT-PCR	gctcttattggataccttgttgg	cttattcgagtcgagtgga
cisd-3.1	RT-PCR	ggaatacggtctgcaagga	catgtgaaccttagttggtagc
cisd-3.2	RT-PCR	acgctgatgtatccaaagc	caactcctgcgtaaactgg
