# Supplementary material for: The cisd gene family regulates physiological germline apoptosis through ced-13 and the canonical cell death pathway in Caenorhabditis elegans
Source: Cell Death Differ. 2018 Apr 17;26(1):162–78. doi: 10.1038/s41418-018-0108-5 (PMC6294797; doi:10.1038/s41418-018-0108-5)
Supplement: Supplementary file 11 — Supplementary figure legends [file 41418_2018_108_MOESM11_ESM.docx]

**Supplementary Figure Legends**

**Figure S1. Multiple protein sequence alignments for members of the CISD family. (a)** Alignment of the *C. elegans* (Ce) CISD-1a, CISD-1b, CISD-3.1 and CISD-3.2 proteins. **(b)** Alignment of the two *cisd-1* isoforms, CISD-1a and CISD-1b, encoded by the *cisd-1* gene. The amino acids deleted in the *tm4993* allele are designated in red and the insertion site for the *pnIs27* allele is noted by a blue triangle. **(c)** Alignment of the CISD-1 isoforms aligned with the human (Hs) mitoNEET/CISD1 and NAF-1/CISD2 proteins. **(d)** Alignment of the CISD-3.1 and CISD-3.2 proteins aligned with the human Miner2/CISD3 protein. **(a-d)** Identical amino acids are in bold. The symbol below the amino acid alignment notes identical amino acids (*), conserved amino acid differences (:), or semi-conserved amino acid differences (.). The CDGSH motif is highlighted in yellow and the transmembrane domain for CISD1/CISD2 is highlighted in blue.

**Figure S2. The *cisd* mRNA levels are reduced by mutation or RNAi of cisd genes.** Shown is the fold change from qRT-PCR assays in **(a)** the *cisd-1(tm4993)* or *cisd-1(pnIs27)* mutants relative to N2 wild-type **(b)** RNAi of *cisd-1*, *cisd-3.1*, *cisd-3.2* relative to N2 wild-type animals fed HT115 control food. Data shown are from 3 independent experiments. Error bars represent standard deviations. The bar indicates p<0.001, one-way ANOVA, Sidak multiple comparisons.

**Figure S3. Disruption of *cisd-1* by RNAi or insertion of the *pnIs27* allele leads to germline abnormalities. (a)** The number of mature oocytes in the *cisd-1(RNAi)* animals is significantly decreased (* indicates P< 0.005, two-tailed unpaired t-test). **(b)** The Mig phenotype is observed in the *cisd-1(pnIs27)* animal (* indicates P< 0.05, two-tailed unpaired t-test). **(c)** The number of mature oocytes in the *cisd-1(pnIs27)* animals is significantly decreased (* indicates P< 0.001, two-tailed unpaired t-test). **(d)** The number of progeny produced by the *cisd-1(pnIs27)* animal is significantly reduced relative to the N2 wild-type animal (* indicates P< 0.0005, two-tailed unpaired t-test). **(e)** Analysis of the gonad using DIC microscopy indicates that the *cisd-1(pnIs27)* animal has a significantly higher number of apoptotic cells relative to the N2 wild-type animal (* indicates P< 0.0001, two-tailed unpaired t-test). For all graphs, the error bar represents standard deviation.

**Figure S4.** **The number of CED-1::GFP marked germ cell corpses is increased in the *cisd-3.1(RNAi)* and *cisd-3.2(RNAi)* animals. (a)** Representative images of the gonad arm of *cisd-3.1(RNAi)*, *cisd-3.2(RNAi)*, or *cisd-3.1(RNAi)*; *cisd-3.2(RNAi)* in the control background (left panel) or the *cisd-1(tm4993)* animal (right panel). The CED-1::GFP fluorescent reporter is shown individually and merged with the DIC image. White arrows point to apoptotic germ cells within the gonad. Scale bar = 20 µm. **(b)** The *cisd-3.1(RNAi)*, *cisd-3.2(RNAi)* and *cisd-3.1(RNAi)*; *cisd-3.2(RNAi)* animal has a significantly higher number of cell death corpses relative to the control animal (bar indicates P < 0.0001, Kruskal-Wallis test, Dunn’s multiple comparisons test). **(c)** The *cisd-3.1(RNAi); cisd-1(tm4993)*, *cisd-3.2(RNAi); cisd-1(tm4993)*, and *cisd-3.1(RNAi)*; *cisd-3.2(RNAi); cisd-1(tm4993)* animals do not have a significantly higher number of cell death corpses relative to the *cisd-1(tm4993)* animal (Kruskal-Wallis test, Dunn’s multiple comparisons test). For b and c, the number of apoptotic germ cell corpses was quantified in animals from three independent experiments for a total of at least thirty animals. Error bar represents standard deviation.

**Figure S5.**  **Disruption of *ced-3* function reduces the number of ACT-5::YFP positive germ cell corpses in the *cisd-1(RNAi)* animal.** The number of apoptotic cells within the gonad were visualized and quantified, using the ACT-5::YFP reporter strain, for the respective animals: control, *ced-3(RNAi)*, *cisd-1(RNAi)*, *cisd-1(RNAi); ced-3(RNAi)*. **(a)** Representative images of the gonad arm are shown; the ACT-5::YFP reporter is shown individually or merged with the DIC image. White arrows point to the apoptotic cells within the gonad. Scale bar = 20 µm. **(b)** The knockdown of *ced-3* using RNAi significantly reduced the number of cell corpses in the gonad of *cisd-1(RNAi)* animals. Relative to control animals the number of cell death corpses within the gonad was significantly different for the *cisd-1(RNAi)* and *ced-3(RNAi)* animals indicating effective RNAi. Identical letters indicate groups with no significant differences; different letters indicate P<0.0001 (one-way ANOVA, Sidak multiple comparisons). The number of apoptotic corpses within the gonad were quantified in animals from three independent experiments for a total of at least thirty animals. Error bar represents standard deviation.

**Figure S6.** **Disruption of the canonical programmed cell death pathway reduces the number of CED-1::GFP-positive germ cell corpses in the *cisd-1(RNAi)* animals.** Using the CED-1::GFP reporter strain the number of apoptotic cells within the gonad was quantified in the *cisd-1(RNAi)* animal with altered *ced-3* function via RNAi. **(a)** Representative images of the gonad arm for control, *ced-3(RNAi)*, *ced-4(n1162), cisd-1(RNAi), cisd-1(RNAi); ced-3(RNAi)*, or *cisd-1(RNAi); ced-4(n1162)* animals. The CED-1::GFP reporter is shown individually or merged with the DIC image. White arrows point to the apoptotic cells within the gonad. Scale bar = 20 µm. **(b)** Knockdown of *ced-3* by RNAi significantly reduced the number of cell corpses in the gonad of *cisd-1(RNAi)* animals. Identical letters indicate groups with no significant differences; different letters indicate P<0.001 (Kruskal-Wallis test, Dunn’s multiple comparisons test) **(c)** The *ced-4(n1162)* mutation significantly reduced the number of cell corpses in the gonad of *cisd-1(RNAi)* animals. Identical letters indicate groups with no significant differences; different letters indicate P<0.0001 (one-way ANOVA, Sidak multiple comparisons). The number of apoptotic corpses within the gonad was quantified in animals from three independent experiments for a total of at least thirty animals. Error bar represents standard deviation.

**Figure S7. The Mig phenotype observed in the *cisd-1(tm4993)* animal is not suppressed by *ced-3(RNAi)* or enhanced by *ced-5(RNAi)* or *ced-10(RNAi)*.** **(a)** Representative images of one gonad arm for N2 wild-type or *cisd-1(tm4993)* one-day old adult hermaphrodites fed RNAi food to target the specified genes. RNAi controls were fed HT115 bacteria. The Mig phenotype was observed in the *ced-5(RNAi)* and *ced-10(RNAi)* animal indicating effective RNAi. The dashed line indicates migration pattern and the black half-moon outlines the DTC. Scale bar = 20 µm. **(b)** The Mig phenotype was significantly higher in the *ced-5(RNAi)* and *ced-10(RNAi)* animal relative to N2 wild-type control animals indicating effective RNAi (Kruskal-Wallis test, Dunn’s multiple comparisons test, bar indicates P< 0.05). The Mig defect observed in the *cisd-1(tm4993)* background is not significantly different in animals with reduced *ced-3,* *ced-5,* or *ced-10* function (grey bars). At least 30 animals from three independent experiments were examined. Error bar represents standard deviation.

**Figure S8.** **Disruption of *egl-1* function by RNAi did not reduce the number of germ cell corpses in the *cisd(tm4993)* animals.** Using the ACT-5::YFP reporter strain, the number of apoptotic cells within the gonad was visualized and quantified in the control, *egl-1(RNAi)*, *cisd-1(tm4993),* and *cisd-1(tm4993); egl-1*(RNAi) animals. (**a)** Representative images of the gonad arm of control, *egl-1(RNAi)*, *cisd-1(tm4993),* and *cisd-1(tm4993); egl-1*(RNAi) animal*.* The ACT-5::YFP reporter is shown individually and merged with the DIC image. White arrows point to apoptotic cells within the gonad. Scale bar = 20 µm. **(b)** Disruption of *egl-1* function by RNAi did not suppress the number of cell death corpses observed in the *cisd-1(tm4993)* animals. Identical letters indicate groups with no significant differences; different letters indicate P<0.0001 (Kruskal-Wallis, Dunn’s multiple comparison test). The number of apoptotic corpses within the gonad were quantified in animals from three independent experiments for a total of at least thirty animals. Error bar represents standard deviation.

**Figure S9.** **The *ced-13(sv32)* mutation reduced the number of ACT-5::YFP-positive germ cell corpses in the *cisd-1(tm4993)* animal.** Using the ACT-5::YFP reporter strain, the number of apoptotic cells within the gonad were visualized and quantified in the control, *ced-13(sv32),* *cisd-1(tm4993),* and *cisd-1(tm4993); ced-13(sv32),* animals. (**a)** Representative images of the gonad arm of control, *ced-13(sv32),* *cisd-1(tm4993),* and *cisd-1(tm4993); ced-13(sv32),* animals*.* The ACT-5::YFP reporter is shown individually and merged with the DIC image. White arrows point to apoptotic cells within the gonad. Scale bar = 20 µm. **(b)** The *ced-13(sv32)* mutation reduced the number of germ cell corpses within the *cisd-1(tm4993)* animals. Identical letters indicate groups with no significant differences; different letters indicate P<0.05 (Kruskal-Wallis, Dunn’s multiple comparison test). Apoptotic germ cell corpses were quantified in animals from three independent experiments for a total of at least thirty animals. Error bar represents standard deviation.
